# Lipid signatures in two functionally selected effluents from clinically relevant postoperative pancreatic fistulas are associated with graded, cell type–specific transcriptional responses

**DOI:** 10.1038/s41598-026-63058-1

**Published:** 2026-07-23

**Authors:** Johannes D. Lettner, Marvin Schwarzer, Simon Lagies, Bernd Kammerer, Stephanie Mewes, Sophia Chikhladze, Stefan Fichtner-Feigl, Geoffroy Andrieux, Dietrich A. Ruess, Uwe A. Wittel

**Affiliations:** 1 Department of General and Visceral Surgery, Center for Surgery, Faculty of Medicine, Medical Center University of Freiburg, University of Freiburg, Freiburg, Germany; 2 German Cancer Consortium (DKTK), Partner Site Freiburg and German Cancer Research Center (DKFZ), Heidelberg, Germany; 3 BIOSS Center of Biological Signaling Studies, University of Freiburg, Freiburg, Germany; 4 Core Competence Metabolomics, Hilde-Mangold-Haus, University of Freiburg, Freiburg, Germany; 5 Spemann Graduate School of Biology and Medicine (SGBM), University of Freiburg, Freiburg, Germany; 6 Institute of Organic Chemistry, University of Freiburg, Freiburg, Germany; 7 Institute of Medical Bioinformatics and Systems Medicine, Faculty of Medicine, Medical Center University of Freiburg, University of Freiburg, Freiburg, Germany

**Keywords:** Postoperative pancreatic fistula, Metabolomics, Transcriptomics, Biochemical risk profiling, Cell biology, Diseases

## Abstract

Clinically relevant postoperative pancreatic fistula (CR-POPF) remains a major cause of morbidity after pancreatoduodenectomy, yet the biochemical composition of postoperative effluents and their effects on anastomotic healing remain poorly understood. We investigated whether CR-POPF effluents exhibit lipolysis-associated lipid signatures and induce defined responses in cellular model systems relevant to anastomotic healing. Drain effluents from 14 patients (7 CR-POPF, 7 non-POPF) underwent GC–MS lipid profiling. Metabolic viability assays (ATP-based readout of cellular stress responses) were performed in peritoneal mesothelial cells and human foreskin fibroblasts as stromal model systems relevant to anastomotic healing, as well as PanC-1 cells. Two CR-POPF effluents associated with reproducible reductions in metabolic viability were functionally selected for transcriptomic profiling. RNA sequencing was performed in mesothelial cells and fibroblasts after exposure to these effluents and monopalmitin. CR-POPF effluents showed coordinated enrichment of long-chain fatty acids and monoacylglycerides. Only a subset reproducibly impaired cellular metabolic viability. Transcriptomic profiling of the two functionally selected CR-POPF effluents revealed a lipid-responsive transcriptional signal across these biologically distinct effluent samples, characterized by induction of ANGPTL4, HMOX1, PLIN2, and PDK4 and consistent with a metabolic and stress-adaptive transcriptional response to lipid exposure. Activation followed a clear gradient (monopalmitin > AES1448 > GR1479) and was more pronounced in mesothelial cells than in fibroblasts. Functionally active CR-POPF effluent samples may carry lipid signals that are associated with a consistent transcriptional signal in peritoneal mesothelial cells and fibroblasts in functionally active effluent samples. The lipid-dependent amplitude and compartment-specific embedding of this signal support a hypothesis-generating framework derived from the two functionally selected CR-POPF effluents.

**Supplementary Information:**

The online version contains supplementary material available at 10.1038/s41598-026-63058-1.

## Introduction

Over the past decades, considerable efforts have focused on risk prediction and perioperative stratification of Clinically relevant postoperative pancreatic fistula (CR-POPF) as one of the most serious complications after pancreatic surgery^1,2^. Although these models identify high-risk patients with increasing accuracy, this has not translated into a substantial reduction in fistula rates or severity, as reflected by the persistently high incidence of CR-POPF, up to 15%, despite major advances in surgical techniques and perioperative care, with ongoing impact on postoperative morbidity and mortality^3–5^.

The biological mechanisms underlying fistula formation and progression, however, remain incompletely understood^4,6,7^. In addition to the well-established mechanical and anatomical risk factors (i.e. soft gland texture, duct size and body mass index), recent studies suggest that locally generated free fatty acids may represent an additional biochemical factor contributing to clinically relevant postoperative pancreatic fistula (CR-POPF)^4,8^. Leakage of pancreatic juice into the peritoneal cavity promotes lipase-mediated intraperitoneal lipolysis, leading to accumulation of free fatty acids measurable in postoperative drain fluid^8^. Experimental studies have shown that saturated free fatty acids such as palmitic acid exert direct cytotoxic effects by inducing oxidative stress, endoplasmic reticulum stress, and pro-inflammatory signaling^9^. This mechanism is analogous to acute pancreatitis, in which lipase-mediated degradation of visceral fat generates free fatty acids that amplify inflammation and parenchymal necrosis^10^. In line with this concept, the International Study Group of Pancreatic Surgery (ISGPS) has identified postoperative pancreatitis as a key component of CR-POPF pathogenesis^11^. Notably, more than half of patients with pancreatic ductal adenocarcinoma (PDAC) exhibit preoperative pancreatic steatosis, suggesting a lipid-rich microenvironment in a substantial proportion of surgical patients^12^. In obese mice, lipase-mediated lipotoxicity converts mild pancreatitis into severe disease, whereas lean animals are protected, highlighting the modifying role of adiposity^13^. Importantly, lipolysis of peripancreatic adipose tissue generates defined cytotoxic lipid species rather than a uniform lipid milieu^8^. Among these, long-chain saturated fatty acids, particularly palmitic acid, arise during lipase-mediated triglyceride breakdown and under hyperlipidemic conditions^14^. Beyond direct lipotoxicity, palmitic acid facilitates inflammasome priming and NF-κB–dependent inflammatory signaling and induces macrophage polarization toward a pro-inflammatory M1 phenotype, promoting cathepsin S–containing exosome release and pyroptotic acinar cell death^15,16^. From a clinical perspective, hyperlipidemia may therefore act as a mechanistic driver of lipotoxic inflammation and innate immune activation within a unified pathogenic framework^14,15^. Nevertheless, direct insights into lipid-driven transcriptional stress responses in cellular compartments that are relevant for anastomotic healing remain limited. It is relevant to note that CR-POPF is indicative of anastomotic healing failure as opposed to primary acinar cell destruction. The regulation of extracellular matrix deposition, scar formation and local immune modulation at the pancreatic anastomosis is mediated by cellular compartments, including fibroblasts and peritoneal mesothelial cells, which are relevant at the peritoneal interface of anastomotic healing. Lipotoxic injury affecting these cellular populations may influence cellular processes involved in tissue repair and compromised anastomotic integrity. This study explores lipid-associated transcriptional responses induced by functionally selected CR-POPF effluents using fibroblast and peritoneal mesothelial cell model systems relevant to postoperative healing.

## Results

### Characteristics of the study sample resource cohort

Samples of a total of 14 patients, seven with clinically relevant postoperative pancreatic fistula (CR-POPF) and seven without (non-POPF) were included. There were no significant differences in baseline demographic and preoperative variables between the groups, including age, sex, BMI, ASA classification, diabetes, and duct width (all *p* > 0.05). Preoperative laboratory values, including serum amylase, lipase and bilirubin, were also similar. Surgical approaches were evenly distributed (50% open, 43% laparoscopic and 7% robotic; *p* = 0.57) and pancreatic texture was soft in 71% of cases, with no significant differences between groups. As expected, based on the ISGPS definition, persistently elevated drain amylase levels were observed in patients classified as CR-POPF. In contrast, serum amylase showed only a transient increase on postoperative day 2 (*p* = 0.007), which may reflect local postoperative inflammation and could be associated with CR-POPF development. Histopathological entities were balanced between groups (*p* = 0.77). Overall complication rates (63%) and severe complications (Clavien–Dindo ≥ III: 43%) did not differ significantly (Table 1).

**Table 1:** Characteristics of the study sample resource cohort. Demographic, surgical, pre-, intra- and postoperative outcomes. Abbreviations: ASA, American Society of Anesthesiologists; BMI, body mass index; IQR, interquartile range; POD, postoperative day; PD, pancreatoduodenectomy; PDAC, pancreatic ductal adenocarcinoma; IPMN, intraductal papillary mucinous neoplasm; AMPAC, ampullary carcinoma; HG, high-grade; LG, low-grade.

	All *n* = 14	CR-POPF = 7	Non-POPF = 7	
Demographic factors				
Age, years, median [IQR]	67.5 [56.8–76]	67 [56–76]	68 [57–79]	0.62
Sex, female, n [%]	4 [28.6%]	4 [57.1]	0 [0]	0.07
BMI, kg/m², median [IQR]	23.7 [22.2–26.4]	23 [21-30.7]	24.4 [22.-25]	0.805
**Pre- and intraoperative variables**				
ASA ≥ III, n [%]	8 [57.1]	5 [71.4]	3 [42.9]	0.405
Diabetes mellitus, yes, n [%]	6 [42.9]	2 [28.6]	4 [57.1]	0.592
History of pancreatitis, yes, n [%]	2 [14.3]	2 [28.6]	0 [0]	0.301
Serum Amylase, U/L, median [IQR]	18.5 [11.5–35.3]	33 [18–36]	12 [6–27]	0.53
Serum Lipase, U/L, median [IQR]	58 [16.8–105]	75 [17–129]	53 [16–67]	0.62
Serum bilirubin, mg/dl, median [IQR]	0.6 [0.3–4.8]	0.4 [0.3–0.6]	0.9 [0.5–9.3]	0.073
Procedure				0.565
Open PD, n [%]	7 [50]	4 [57.1]	3 [42.9]	
Lap PD, n [%]	6 [42.9]	3 [42.9]	3 [42.9]	
Robotic PD, n [%]	1 [7.1]	0 [0]	1 [14.3]	
Duct width, mm, median [IQR]	3[ 3–4]	3 [3–4]	4 [3–7]	0.318
Pancreatic texture, soft, n [%]	10 [71.4]	6 [85.7]	4 [57.1]	0.559
**Postoperative Outcomes**				
Serum Amylase POD1, U/L, median [IQR]	39.5 [6-55.5]	40 [38–72]	6 [3–42]	0.165
Serum Amylase POD2, U/L, median [IQR]	29.5 [5.8–73]	70 [28–138]	6 [2–31]	**0.007**
Serum Amylase POD3, U/L, median [IQR]	18 [6.3–68.8]	65 [24–98]	7 [2–13]	**0.001**
Drain Amylase POD1, U/L, median [IQR]	248 [18.8–1900]	1792 [1115–5084]	24 [3–36]	**0.001**
Drain Amylase POD2, U/L, median [IQR]	669 [16-3952]	3046 [1287–6935]	17 [13–104]	**0.001**
Drain Amylase POD3, U/L, median [IQR]	566.5 [11-2121.3]	1947 [1056–4440]	11 [4–47]	**0.001**
Fistula Grade				**< 0.001**
B, n [%]	6 [42.9]	6 [85.7]	0 [0]	
C, n [%]	1 [7.1]	1 [14.3]	0 [0]	
Histopathology				0.771
PDAC Head, n [%]	5 [35.7]	2 [28.6]	3 [42.9]	
AMPAC, n [%]	4 [28.6]	2 [28.6]	2 [28.6]	
HG IPMN, n [%]	2 [14.3]	1 [14.3]	1 [14.3]	
LG IPMN, n [%]	2 [14.3]	1 [14.3]	1 [14.3]	
Chronic pancreatitis, n [%]	1 [7.1]	1 [14.3]	0 [0]	
Any Complications within 30d, yes, n [%]	12 [63.2]	7 [100]	5 [71.4]	0.462
Clavien–Dindo ≥ III, n [%]	6 [42.9]	4 [57.1]	3 [42.9]	0.225

### CR-POPF effluents are enriched in triglyceride-derived saturated lipids

**Fig. 1:**
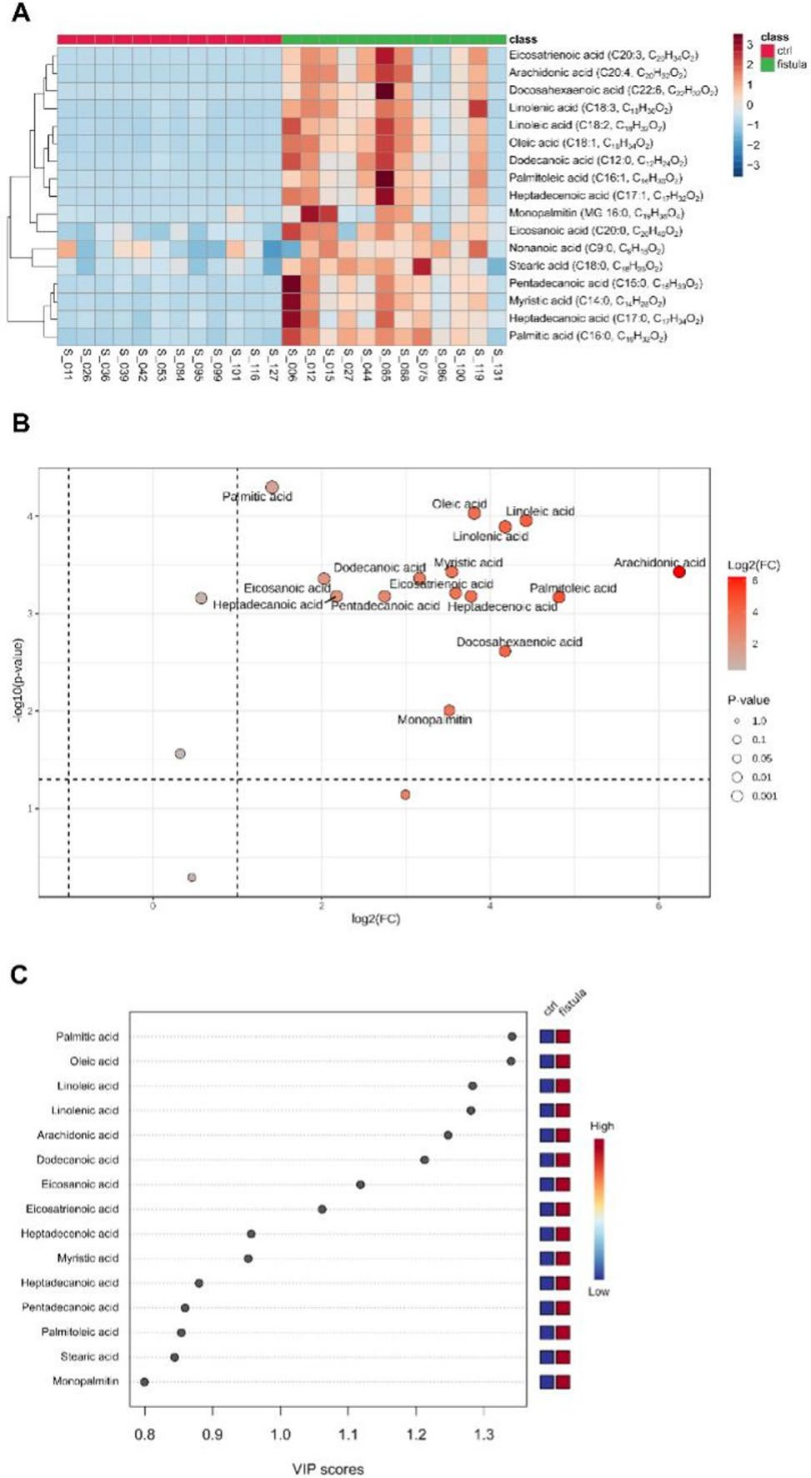
CR-POPF effluents exhibit enrichment of triglyceride-derived saturated lipids. (**A**) Heatmap of significantly altered lipid species identified by GC–MS (FDR < 0.05) comparing CR-POPF and non-POPF effluents. Samples are grouped by clinical class. CR-POPF effluents demonstrate coordinated enrichment of long-chain saturated fatty acids and monoacylglycerides. (**B**) Volcano plot depicting log₂ fold change versus –log₁₀ adjusted p-value for detected lipid species. Multiple long-chain saturated fatty acids and monopalmitin are significantly enriched in CR-POPF effluents (FDR threshold indicated). (**C**) Variable importance in projection (VIP) scores derived from supervised partial least squares discriminant analysis (PLS-DA). Palmitic acid, stearic acid, and monopalmitin rank among the strongest contributors to class separation between CR-POPF and non-POPF effluent.

Gas chromatography–mass spectrometry (GC–MS) was performed to quantify lipolysis-associated lipid species, including long-chain fatty acids and monoacylglycerides, in postoperative drain effluents from CR-POPF and non-POPF patients. The heatmap of significantly altered lipid species (FDR < 0.05) demonstrated a clear segregation between CR-POPF and control effluents (Fig. 1A). CR-POPF samples were characterized by enrichment of multiple long-chain saturated fatty acids, including palmitic acid (C16:0), stearic acid (C18:0) as well as monoacylglycerides such as monopalmitin. This pattern was not driven by isolated outliers but reflected a consistent lipid signature across the fistula group. Differential analysis at the individual metabolite level confirmed these shifts (Fig. 1B). Multiple lipid species exhibited significant fold changes, with several long-chain fatty acids and monopalmitin showing both high effect size and statistical significance. Among the altered metabolites, monopalmitin displayed one of the most distinct separations between CR-POPF and non-POPF effluents, indicating enrichment of triglyceride breakdown products within the fistula-associated biochemical milieu. Monopalmitin levels were consistently higher in CR-POPF effluents compared with controls (Figure S1). To quantify the contribution of individual metabolites to class separation, supervised partial least squares discriminant analysis (PLS-DA) was performed, and variable importance in projection (VIP) scores were calculated (Fig. 1C, S2, S3). Palmitic acid, stearic acid, and monopalmitin ranked among the highest discriminators between CR-POPF and control samples. The concordance between univariate significance, fold-change magnitude, and multivariate importance supported the robustness of these species as central components of the CR-POPF lipid profile. Quality control assessment demonstrated analytical stability, with low rates of missing values (< 10%) and consistent signal intensity across blank, control, and QC samples (Figure S4). Correlation analysis further revealed positive associations among long-chain saturated fatty acids, including palmitic, stearic, heptadecanoic, and myristic acid, indicating coordinated release of triglyceride-derived lipid species rather than isolated metabolite accumulation (Figure S5).

Together, these data support the presence of a lipolysis-associated lipid signature within the analyzed effluent samples, characterized by enrichment of long-chain saturated fatty acids and monoacylglycerides. Based on their statistical significance, multivariate relevance, and biochemical plausibility, palmitic acid, stearic acid, and monopalmitin were selected for subsequent functional experiments.

### Fatty acid challenge across fibroblast, peritoneal mesothelial, and carcinoma cell types

To establish sublethal concentration ranges for subsequent experiments, HFF, peritoneal and pancreatic tumor cells were exposed to increasing concentrations of palmitic acid, stearic acid, and monopalmitin (0.1, 0.2, 0.5 mM). Two-way ANOVA revealed significant main effects of concentration and fatty acid type as well as significant interactions in all three cell types (HFF: F(8, 60) = 2.6, *p* = 0.0138; peritoneal: F(8, 57) = 12.8, *p* < 0.0001; PanC-1: F(8, 57) = 3.4, *p* = 0.0027). Repeated measures two-way ANOVA on normalized viability confirmed robust effects of both factors and their interaction (HFF: F(6, 36) = 44; peritoneal: F(6, 45) = 125; PanC-1: F(6, 45) = 34; all *p* < 0.0001). Across all models, concentration explained roughly one-third to one-half of the total variance, with the strongest contribution from fatty acid type. Viability decreased consistently with rising concentrations, most prominently at 0.5 mM. Palmitic acid caused the greatest loss of viability in all cell types, while stearic acid and monopalmitin produced only minor effects up to 0.2 mM (data not shown). Collectively, these results defined 0.2 mM as a standardized upper sublethal reference concentration for subsequent effluent-exposure and transcriptomic profiling experiments. Direct quantitative comparison to lipid concentrations in patient-derived effluents is not feasible based on the available metabolomic data.

### Only a subset of CR-POPF effluents reproducibly impairs metabolic viability

**Fig. 2:**
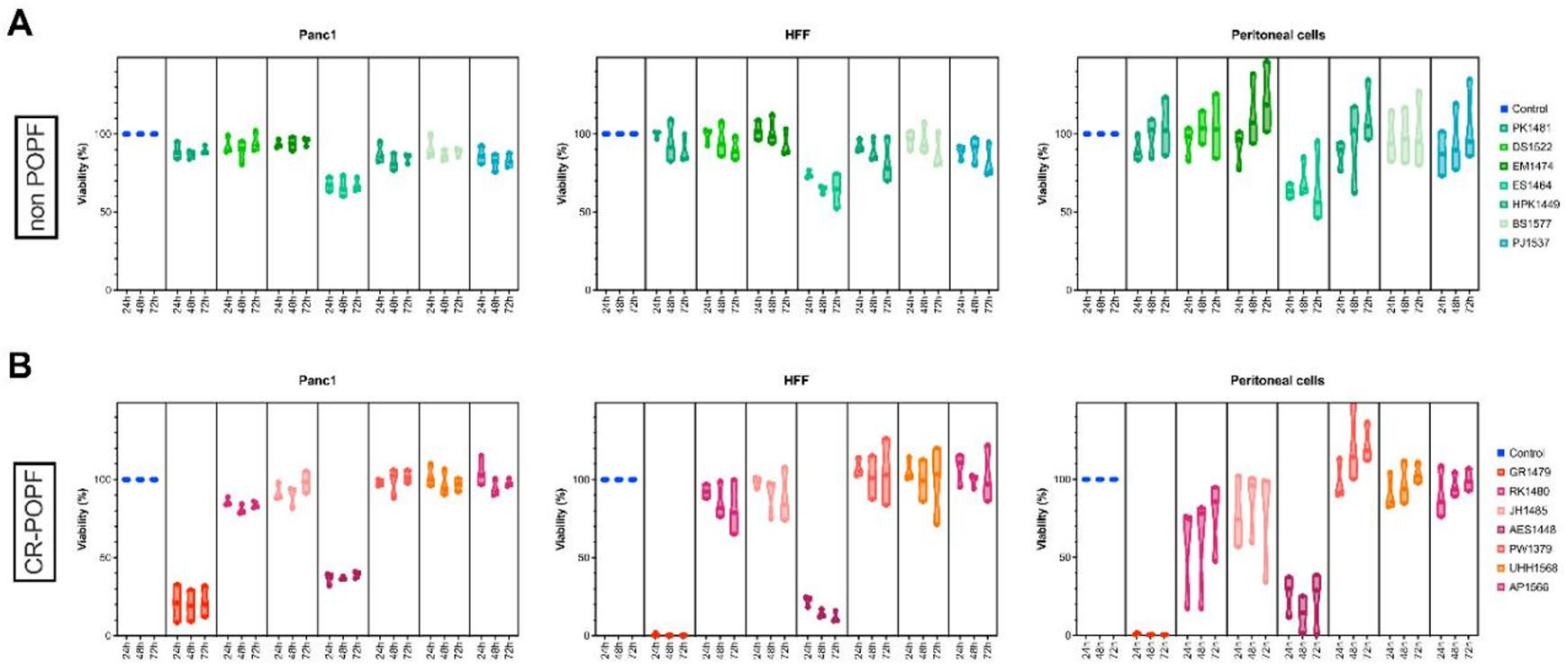
Functional screening identifies two functionally active CR-POPF effluents for subsequent transcriptomic profiling. (**A**) ATP-based metabolic viability following exposure to non-POPF effluents in PanC-1 cells (epithelial reference model), human foreskin fibroblasts (HFF), and primary peritoneal mesothelial cells. Across all cell types, non-POPF effluents did not induce relevant reductions in metabolic activity compared with medium controls. (**B**) ATP-based metabolic viability following exposure to CR-POPF effluents in the same models. While responses varied between individual samples, a subset of CR-POPF effluents induced marked, treatment-dependent reductions in ATP-based metabolic viability, most prominently in cellular compartments (fibroblasts and peritoneal mesothelial cells). AES1448 and GR1479 demonstrated the strongest and most reproducible cellular viability impairment and were selected for subsequent transcriptomic profiling. Viability is expressed relative to medium control. Statistical analysis was performed using two-way ANOVA; detailed statistics are provided in the Results section.

In PanC-1, exposure to non-fistula effluents did not induce a relevant reduction in metabolic viability or an increase in membrane integrity–based cytotoxicity. Two-way ANOVA of absolute metabolic viability values showed significant effects of time (F(2,48) = 48.6, *p* < 0.0001) and treatment (F(7,48) = 7.1, *p* < 0.0001), but no interaction (F(14,48) = 0.15, *p* = 0.99). Analyses of normalized viability confirmed that variability was primarily driven by differences between individual non-fistula effluents (F(7,48) = 34.9, *p* < 0.0001), with no relevant time dependence. Overall, the ATP-based metabolic activity of pancreatic carcinoma cells, as assessed by CellTiter-Glo, remained close to that of the medium controls following exposure to non-fistula effluents. In contrast, exposure to CR-POPF effluents resulted in significant reductions in metabolic activity that varied between samples. Two-way ANOVA revealed significant main effects of time (F(2, 48) = 23.5, *p* < 0.0001) and treatment (F(7, 48) = 25.6, *p* < 0.0001), with no significant interaction effect (F(14, 48) = 0.48, *p* = 0.93). Treatment accounted for nearly two-thirds of the total variance and dominated the response pattern. Analyses of normalized metabolic activity confirmed that treatment was the only significant contributing factor (F(7, 48) = 229.7, *p* < 0.0001). Among the CR-POPF effluents, AES1448 and GR1479 were associated with the most significant reductions in metabolic activity at the analyzed time points and were therefore selected as functionally active effluents for subsequent transcriptomic analyses (Fig. 2).

Following exposure to non-fistula effluents, fibroblasts displayed stable ATP-based metabolic activity. A two-way ANOVA of the absolute metabolic activity values revealed significant main effects of time (F(2, 48) = 245.5, *p* < 0.0001) and treatment (F(7, 48) = 8.6, *p* < 0.0001), and no significant interaction effect (F(14, 48) = 0.82, *p* = 0.65). Analyses of normalized metabolic activity confirmed this pattern, with significant effects of time (F(2, 48) = 5.6, *p* = 0.0066) and treatment (F(7, 48) = 13.8, *p* < 0.0001). Metabolic activity remained close to medium controls across non-fistula effluents. In contrast, exposure to CR-POPF effluents resulted in pronounced, treatment-dependent reductions in metabolic activity. A two-way ANOVA revealed significant main effects of time (F(2, 48) = 34.0, *p* < 0.0001) and treatment (F(7, 48) = 32.5, *p* < 0.0001), but no significant interaction effect (F(14, 48) = 1.64, *p* = 0.10). Treatment explained approximately 62% of the total variance. In normalized analyses, treatment remained the only significant source of variation (F(7, 42) = 261.4, *p* < 0.0001). Among CR-POPF effluents, AES1448 and GR1479 were associated with the most pronounced and consistent reductions in metabolic activity across replicates and analyzed points of time (Fig. 2). Peritoneal cells exhibited stable ATP-based metabolic activity following exposure to non-fistula effluents. Two-way ANOVA of absolute metabolic activity values showed modest but significant main effects of time (F(2,48) = 8.1, *p* = 0.0009) and treatment (F(7,48) = 3.0, *p* = 0.0107), without a significant interaction (F(14,48) = 0.90, *p* = 0.998). Analyses of normalized metabolic activity confirmed these mild time-dependent changes (time F(2,48) = 3.8, *p* = 0.030; treatment F(7,48) = 5.1, *p* = 0.0002), with no effluent causing a pronounced reduction in metabolic activity relative to controls. In contrast, exposure to CR-POPF effluents resulted in a distinctly treatment-dependent response in peritoneal mesothelial cells. Two-way ANOVA demonstrated significant main effects of time (F(2,48) = 4.6, *p* = 0.015) and treatment (F(7,48) = 19.8, *p* < 0.0001), while the interaction remained non-significant (F(14,48) = 0.38, *p* = 0.97). Treatment explained nearly 70% of the total variance. Consistent with findings in fibroblasts and pancreatic carcinoma cells, the effluents AES1448 and GR1479 were associated with the most pronounced and reproducible reductions in metabolic activity across the analyzed time points, motivating their selection for subsequent transcriptomic analyses (Fig. 2). Taken together, ATP-based metabolic activity assays indicated that non-fistula effluents were largely inert across all examined cell types, whereas only a subset of CR-POPF effluents induced consistent, treatment-dependent reductions in ATP-based metabolic activity as an integrative stress-associated readout. Based on these findings, effluents AES1448 and GR1479, together with monopalmitin as a defined lipid reference stimulus, were selected for transcriptomic profiling in fibroblasts and peritoneal mesothelial cells. These transcriptomic analyses therefore represent functionally selected effluents rather than the complete spectrum of CR-POPF samples.

### RNA sequencing and transcriptomic analysis

To explore transcriptional programs engaged by CR-POPF effluents (AES1448, GR1479) and monopalmitin, mRNA sequencing was performed in five evaluable non-malignant peritoneal mesothelial cell preparations (P1–P6) and human foreskin fibroblasts (HFF) as a standardized fibroblast model following exposure to AES1448, GR1479, monopalmitin (0.2 mM), or medium control. Importantly, these transcriptomic analyses were performed with functionally selected, biologically active effluents in an exploratory, hypothesis-generating endeavor, and need to be interpreted as mechanistic exemplars rather than representative of the full spectrum of CR-POPF effluents. Accordingly, all subsequent transcriptomic analyses refer specifically to these functionally selected effluents.

**Fig. 3:**
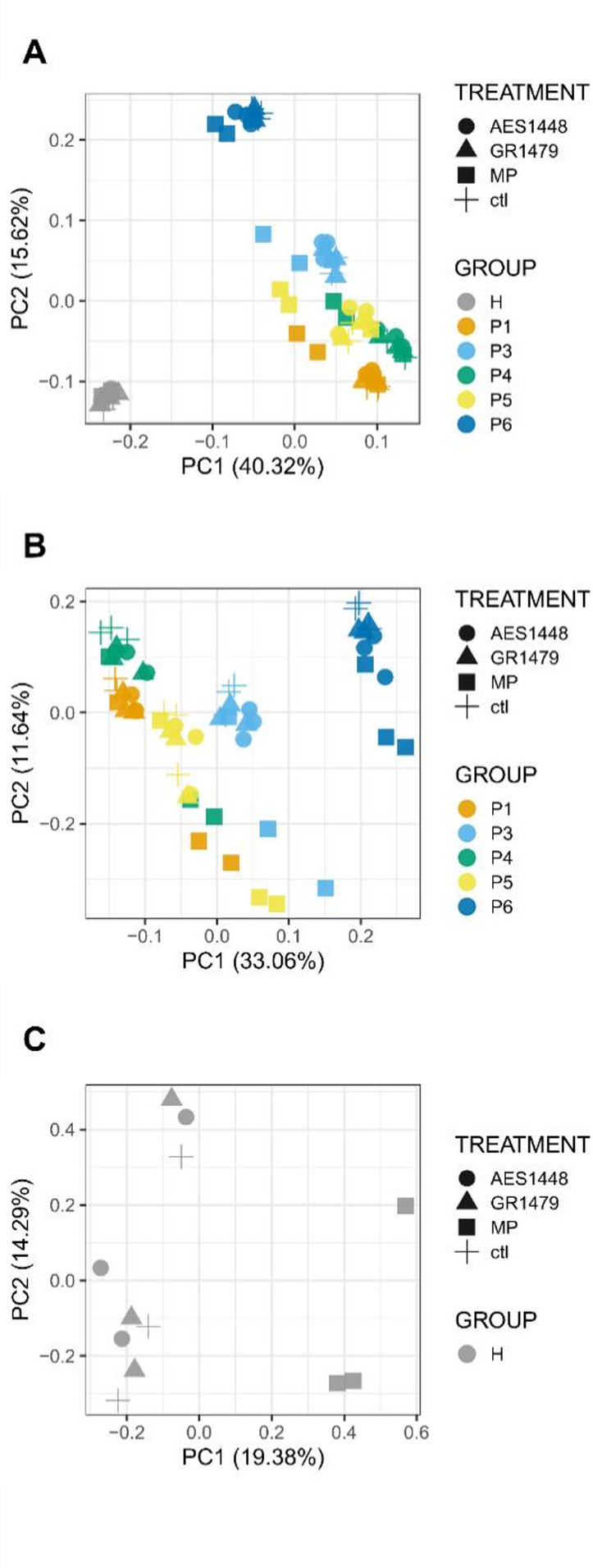
Stimulus-induced transcriptional displacement is graded and cell type–dependent. (**A**) Principal component analysis (PCA) across all samples, including peritoneal mesothelial cell preparations (P1–P6) and human foreskin fibroblasts (HFF) exposed to AES1448, GR1479, monopalmitin (MP, 0.2 mM), or medium control (ctl). One mesothelial preparation (P2) was excluded from analysis because of technical failure during sample processing. PC1 (40.32%) primarily separates samples by cellular origin, clearly distinguishing mesothelial cells from fibroblasts. Within this structure, monopalmitin induces the most pronounced displacement from control and effluent-treated conditions. Biological replicates cluster tightly within each preparation. (**B**) PCA restricted to peritoneal mesothelial cell preparations. PC1 (33.06%) reflects stimulus-associated variance. Monopalmitin-treated samples separate clearly from control conditions, whereas AES1448 and GR1479 produce moderate and partially overlapping transcriptional shifts. Relative positioning of effluent-treated samples indicates partially shared but non-identical transcriptional responses compared with monopalmitin exposure. (**C**) PCA restricted to human foreskin fibroblasts (HFF). PC1 (19.38%) captures stimulus-dependent variance. Monopalmitin induces the strongest displacement, while AES1448 and GR1479 cluster closer to controls. Overall transcriptional dispersion is reduced compared with peritoneal mesothelial cells, indicating more limited stimulus-associated transcriptional separation in fibroblasts.

Principal component analysis across all cell types and conditions revealed that the dominant source of variance was attributable to cell preparation and donor background (Fig. 3A). Samples segregated primarily according to cellular origin, clearly separating peritoneal mesothelial cells from fibroblasts along PC1 (40.32%). Within this global structure, stimulus-dependent shifts were evident but secondary to donor- and cell-type–associated variance. Notably, monopalmitin exposure consistently displaced samples away from control and effluent-treated conditions, indicating induction of a distinct lipid-responsive transcriptional state. In contrast, AES1448 and GR1479 clustered closer to medium controls across preparations, suggesting comparatively moderate transcriptional perturbation by the two selected fistula effluents. Biological replicates formed compact clusters within each preparation, demonstrating reproducibility of stimulus-dependent transcriptional responses. When analysis was restricted to peritoneal mesothelial cells (Fig. 3B), donor-specific effects remained visible but stimulus-dependent structure became more pronounced.

Monopalmitin exposure induced the largest displacement along PC1 (33.06%), separating clearly from control and effluent-treated samples. AES1448 and GR1479 formed partially overlapping clusters that were positioned closer to controls, indicating moderate and qualitatively similar transcriptional shifts. Overall dispersion was broader in peritoneal mesothelial cells compared to fibroblasts, consistent with increased transcriptional responsiveness of these cellular compartments relevant to anastomotic healing.

In fibroblasts analyzed separately (Fig. 3C), overall variance was lower and cluster separation was more compact. Again, monopalmitin induced the most distinct shift along PC1 (19.38%). AES1448 and GR1479 clustered near controls and showed limited separation from each other. Replicates remained tightly grouped, indicating consistent but comparatively restrained transcriptional responses. Relative to peritoneal mesothelial cells, fibroblasts exhibited a narrower transcriptional dispersion across all conditions. These findings suggest that lipid exposure may contribute to a shared component of the transcriptional response, whereas the selected pancreatic fistula effluents may additionally be associated with effluent-specific transcriptional patterns within the analyzed conditions.

**Fig. 4:**
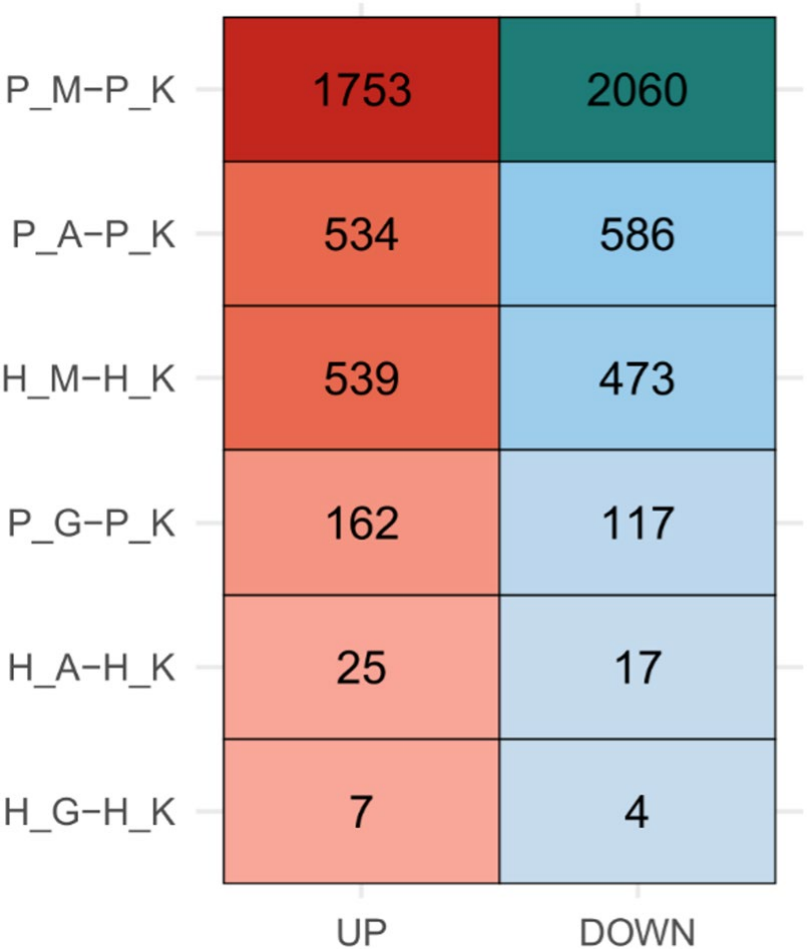
Number of significantly upregulated (UP) and downregulated (DOWN) genes per stimulus relative to medium control in peritoneal mesothelial cells (P) and human foreskin fibroblasts (H). Monopalmitin (M), AES1448 (**A**), and GR1479 (G) are shown. DEG counts are higher in peritoneal mesothelial cells compared with fibroblasts across conditions.

In order to quantify the stimulus-dependent transcriptional amplitude, differential expression analysis was performed for each stimulus relative to the medium control within peritoneal mesothelial cells and fibroblasts (Fig. 4). Across all contrasts, monopalmitin induced the highest number of differentially expressed genes (DEGs). In peritoneal mesothelial cells, monopalmitin resulted in 1.753 upregulated and 2.060 downregulated transcripts compared to the control, whilst in fibroblasts, monopalmitin exposure yielded 539 upregulated and 473 downregulated genes. AES1448 induced an intermediate transcriptional response. In peritoneal cells, 534 genes were upregulated and 586 downregulated, and in fibroblasts, AES1448 affected 25 upregulated and 17 downregulated transcripts. GR1479 elicited the lowest DEG counts. In peritoneal mesothelial cells, 162 genes were upregulated and 117 downregulated, whereas in fibroblasts, only 7 upregulated and 4 downregulated genes were detected. Overall, DEG burden followed a consistent gradient across both cell types, with monopalmitin followed by AES1448 and GR1479. In addition, peritoneal mesothelial cells exhibited substantially higher DEG counts than fibroblasts across all stimuli.

### The two functionally selected CR-POPF effluents and monopalmitin are associated with a shared lipid-responsive transcriptional signal

**Fig. 5:**
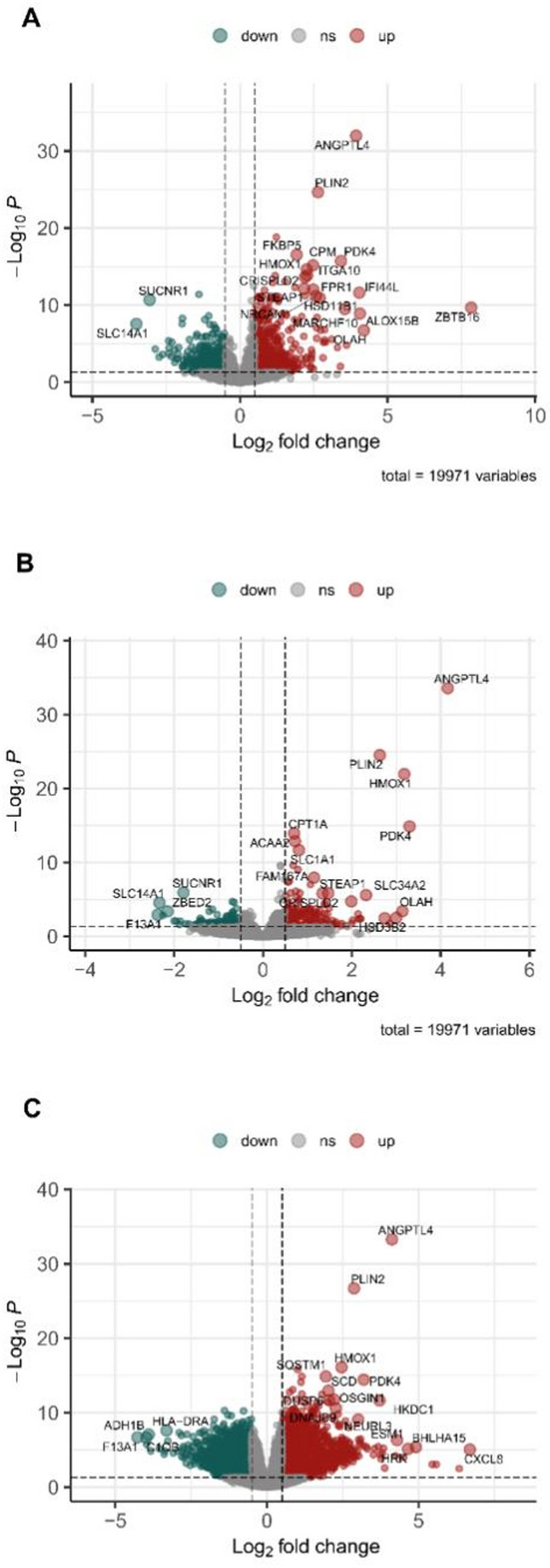
The two functionally selected CR-POPF effluents and monopalmitin are associated with a shared lipid-responsive transcriptional signal in mesothelial cells under the analyzed conditions. (**A**) Cumulative volcano plot of differential gene expression in peritoneal mesothelial cells following exposure to AES1448 compared with control conditions. Data represent integrated results across six independent cell preparations. Red: significantly upregulated genes; green: significantly downregulated genes; grey: not significant (FDR < 0.05). (**B**) Cumulative volcano plot for GR1479 compared with control. The gene-level distribution largely overlaps with AES1448, indicating a shared transcriptional core with moderate amplitude. (**C**) Cumulative volcano plot for monopalmitin (0.2 mM) compared with control. Monopalmitin induces a broader and higher-amplitude distribution of regulated genes, consistent with amplification of the shared lipid-responsive program.

The following analyses are based on functionally selected CR-POPF effluents and should therefore be interpreted as mechanistic exemplars rather than as representative of the full spectrum of CR-POPF effluents. To resolve stimulus-specific gene-level effects across preparations, cumulative volcano plots were generated for AES1448, GR1479, and monopalmitin by integrating all six peritoneal mesothelial cell preparations (Fig. 5). AES1448 exposure resulted in consistent upregulation of ANGPTL4 across all preparations, representing the most significant and highest effect-size signal. Additional recurrently upregulated genes included PLIN2, PDK4, OLAH, ALOX15B, APOB, and HSD11B1. Stress-associated transcripts such as HMOX1, STEAP1, FKBP5, and IFI44L were also significantly induced. Several stromal and adhesion-associated genes, including CPM, ITGA10, NRCAM, and CDH20, were reproducibly modulated. Among downregulated transcripts, SUCNR1 and SLC14A1 were consistently suppressed. Overall, AES1448 induced a focused and reproducible transcriptional pattern with a predominance of metabolic and stress-associated genes (Fig. 5A). GR1479 elicited a highly similar gene-level profile. ANGPTL4 again represented the dominant upregulated transcript. PLIN2, PDK4, CPT1A, ACAA2, and OLAH were recurrently increased across preparations. HMOX1 and STEAP1 were also significantly induced. Downregulated genes included SUCNR1, SLC14A1, and F13A1. The overall distribution and identity of regulated genes largely overlapped with AES1448, with differences mainly in effect magnitude rather than gene composition (Fig. 5B). In contrast, monopalmitin exposure resulted in a broader and higher-amplitude distribution of significantly regulated genes. ANGPTL4 and PLIN2 remained the most prominent upregulated transcripts. Additional strongly induced genes included PDK4, HKDC1, SCD, HMOX1, SQSTM1, OSGIN1, DUSP6, and DNAJB9. Notably, a distinct set of chemokine-associated transcripts, including CXCL8, was prominently upregulated at the transcript level. Downregulated genes included C1QB, HLA-DRA, F13A1, and ADH1B (Fig. 5C). Compared with AES1448 and GR1479, monopalmitin affected a larger number of genes and showed a broader dispersion along both effect size and statistical significance axes. To compare stimulus-dependent transcriptional responses across conditions within fibroblasts, cumulative volcano plots were generated for AES1448, GR1479, and monopalmitin versus control (Figure S6A–C). In fibroblasts, AES1448 induced a limited but coherent set of differentially expressed genes (Figure S6A). ANGPTL4 represented the most significantly upregulated transcript. Additional upregulated genes included PDK4, FKBP5, HMOX1, and ZBTB16, consistent with a metabolic and stress-associated response. Downregulated genes were fewer and of lower amplitude compared with peritoneal mesothelial cells. GR1479 elicited a similarly moderate transcriptional response (Figure S6B). ANGPTL4 was again the dominant upregulated gene, accompanied by HMOX1 and PDK4. Overall effect sizes and numbers of significantly altered genes were comparable to AES1448 and markedly lower than observed in peritoneal mesothelial cells. Monopalmitin exposure resulted in a broader and higher-amplitude transcriptional response in fibroblasts. Strong upregulation of ANGPTL4, PLIN2, PDK4, HMOX1, and SLC16A6 were observed, together with additional transcripts including IL6 and IL24 (Figure S6C). The number and magnitude of regulated genes exceeded those induced by AES1448 or GR1479 in fibroblasts, although the overall transcriptional dispersion remained lower than in peritoneal mesothelial cells. Across all stimuli, ANGPTL4 was the most consistently induced transcript. AES1448 and GR1479 displayed largely overlapping gene-level patterns, whereas monopalmitin showed the same core signature at higher amplitude. In line with the DEG overview, both the number and magnitude of regulated transcripts were greater in peritoneal mesothelial cells than in fibroblasts across conditions.

### Gene set enrichment reveals a shared lipid-responsive program with graded activation across different cell types

**Fig. 6:**
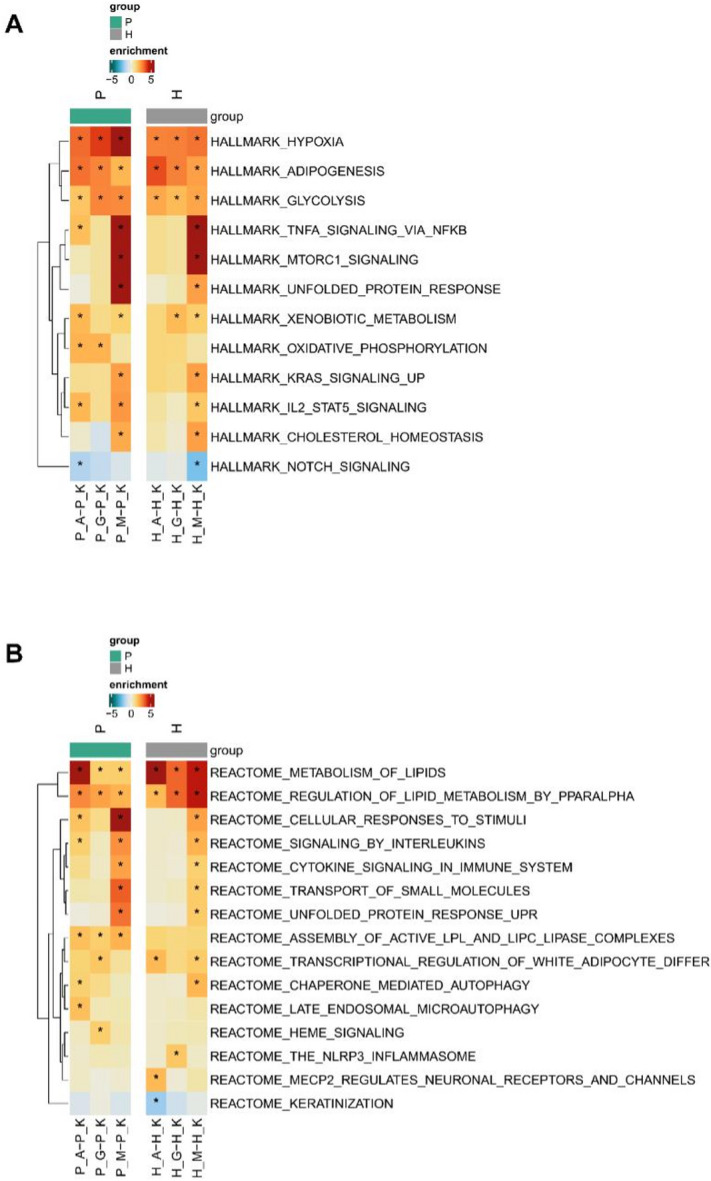
Gene set enrichment reveals a shared lipid-associated program with graded activation and compartment-specific differences. (**A**) Hallmark gene set enrichment across stimuli and cell types. Heatmap of normalized enrichment scores (NES) in peritoneal mesothelial cells (P) and fibroblasts (H) after AES1448 (A), GR1479 (G), or monopalmitin (M) versus control (K). Across conditions, metabolic and stress-associated Hallmark programs were enriched, with highest magnitude under monopalmitin. Peritoneal mesothelial cells exhibited comparatively stronger stress- and immune-associated enrichment than fibroblasts. (**B**) Reactome pathway enrichment across stimuli and cell types which may contribute to anastomotic healing. Heatmap showing normalized enrichment scores for selected Reactome pathways in peritoneal mesothelial cells (P) and fibroblasts (H) following AES1448, GR1479, and monopalmitin exposure versus control. All stimuli were associated with enrichment of lipid metabolism–related pathways, with strongest enrichment observed for monopalmitin. Peritoneal mesothelial cells demonstrated comparatively stronger enrichment of immune-related transcriptional pathway signatures, whereas fibroblasts showed greater representation of lipid-processing and proteostasis-associated pathways.

To assess whether the stimulus-dependent differences in transcriptional amplitude and gene-level signatures reflected coordinated biological programs, we performed gene set enrichment analysis (GSEA) on pooled differential expression contrasts. Hallmark gene set enrichment analysis revealed a consistent pathway architecture across stimuli and cell types (Fig. 6). In both peritoneal mesothelial cells and fibroblasts, all three stimuli were associated with coordinated enrichment of HALLMARK_HYPOXIA, HALLMARK_ADIPOGENESIS, HALLMARK_TNFA_SIGNALING_VIA_NFKB, and HALLMARK_MTORC1_SIGNALING, representing the central transcriptional framework. In peritoneal mesothelial cells, this core program was accompanied by pronounced enrichment of HALLMARK_UNFOLDED_PROTEIN_RESPONSE and reproducible reduction of HALLMARK_NOTCH_SIGNALING. In fibroblasts, the same four hallmark signatures predominated, with relatively stronger enrichment of HALLMARK_GLYCOLYSIS and comparatively lower magnitude of HALLMARK_UNFOLDED_PROTEIN_RESPONSE (Fig. 6A).

Across stimuli, enrichment strength followed a consistent gradient (MP > AES > GR). Monopalmitin showed the highest normalized enrichment scores across hallmark gene sets, whereas AES1448 and GR1479 exhibited highly overlapping pathway profiles that differed mainly in magnitude rather than identity. Overall, hallmark analysis supports a shared transcriptional core program across stimuli and cell types, with quantitative differences in enrichment intensity and relative pathway engagement (Figure S7, S8A–C).

To further resolve the functional architecture underlying the shared hallmark programs, we next performed gene set enrichment analysis using the Reactome pathway collection. Reactome pathway analysis demonstrated that AES, GR, and MP were consistently associated with enrichment of REACTOME_METABOLISM_OF_LIPIDS, REACTOME_REGULATION_OF_LIPID_METABOLISM_BY_PPARALPHA, and REACTOME_CELLULAR_RESPONSES_TO_STIMULI across both stromal cell types (Fig. 6B). In addition, REACTOME_TRANSPORT_OF_SMALL_MOLECULES and REACTOME_UNFOLDED_PROTEIN_RESPONSE_UPR showed recurrent enrichment. The magnitude of enrichment followed a graded pattern (MP > AES > GR), particularly within lipid metabolism–associated pathways. Despite this shared lipid-centered enrichment profile, pathway distribution differed between cell types. Peritoneal mesothelial cells exhibited comparatively stronger enrichment of immune-associated pathways, including REACTOME_SIGNALING_BY_INTERLEUKINS, REACTOME_CYTOKINE_SIGNALING_IN_IMMUNE_SYSTEM, and REACTOME_THE_NLRP3_INFLAMMASOME. In contrast, fibroblasts showed relatively greater representation of REACTOME_ASSEMBLY_OF_ACTIVE_LPL_AND_LIPC_LIPASE_COMPLEXES, REACTOME_TRANSCRIPTIONAL_REGULATION_OF_WHITE_ADIPOCYTE_DIFFERENTIATION, and REACTOME_CHAPERONE_MEDIATED_AUTOPHAGY. These differences suggest that, despite a shared lipid-centered core response, peritoneal mesothelial cells may engage additional immune-associated signaling layers, whereas fibroblasts may display comparatively stronger enrichment of lipid-processing and proteostasis-related pathways.

To further contextualize the stimulus-induced transcriptional programs beyond pathway-level enrichment, additional MSigDB collections were interrogated, yielding complementary insights into biological processes, immune activation states, cell type resemblance, and perturbation-associated stress signatures. GO Biological Process enrichment analysis confirmed a stress- and metabolism-centered transcriptional architecture across cell types, mainly under influence of monopalmitin, characterized by coordinated activation of endoplasmic reticulum stress, proteasomal protein catabolism, and lipid metabolic processes (Figure S9). To contextualize this framework within established immune activation states, enrichment against curated ImmunesigDB signatures demonstrated partial overlap with monocyte- and macrophage-derived transcriptional programs, particularly in peritoneal mesothelial cells (Figure S10). C8 cell type signature enrichment analysis further indicated that peritoneal mesothelial cells displayed stronger resemblance to immune-associated profiles, whereas fibroblasts retained comparatively greater representation of stromal and mesenchymal gene sets (Figure S11). Finally, interrogation of chemical and genetic perturbation (C2-CGP) datasets revealed concordant enrichment of hypoxia- and NRF2-associated stress signatures across both stromal cell types, with the strongest magnitude observed following monopalmitin exposure (Figure S12).

Despite reproducible reductions in ATP-based metabolic viability following exposure to selected CR-POPF effluents, gene set enrichment analysis did not demonstrate consistent enrichment of canonical apoptosis, cell cycle arrest, or proliferation-associated transcriptional programs across conditions. These pathway-level findings were not complemented by protein-level cytokine measurements or downstream functional assays. Instead, enrichment patterns were dominated by metabolic and stress-associated pathways, including hypoxia, mTORC1 signaling, unfolded protein response, TNF/NF-κB signaling, and NRF2-related oxidative stress signals. This pattern was observed in both cellular compartments and followed the same amplitude gradient (monopalmitin > AES1448 > GR1479). These findings should be interpreted within the context of the two functionally selected fistula effluents and do not imply generalizability across all CR-POPF samples.

## Discussion

### Principal findings

Our data suggest that effluents from clinically relevant pancreatic fistulas may represent biochemically heterogeneous lipid environments that are associated with specific transcriptional responses in peritoneal mesothelial cells and fibroblasts. A lipid-centered transcriptional signal was consistently observed across the two functionally selected, biologically distinct CR-POPF effluents and the lipid species monopalmitin. Importantly, this transcriptional framework is derived from two functionally selected CR-POPF effluents and should therefore be interpreted as a mechanistic exemplar rather than a class-wide signature of CR-POPF effluents and may reflect transcriptional adaptation to lipolysis-derived lipid exposure rather than overt injury in cellular compartments relevant to anastomotic healing. This response followed a quantitative gradient (monopalmitin > AES1448 > GR1479), with peritoneal mesothelial cells exhibiting broader and higher-amplitude activation than fibroblasts. Notably, monopalmitin was associated with stronger immune-embedded signaling, suggesting that increasing lipid burden may amplify inflammatory transcriptional components. Transcriptional programs were dominated by metabolic and stress-adaptive pathways. Although selected CR-POPF effluents reduced ATP-based metabolic viability, we did not observe consistent enrichment of canonical apoptosis, proliferation arrest, or cell death–associated programs. This dissociation may indicate that reduced metabolic viability reflects stress-induced metabolic constraint rather than transcriptionally executed cell death. Thus, the ATP-based metabolic viability phenotype is consistent with graded metabolic stress integration rather than overt cytotoxic collapse. These observations are based on ATP-based metabolic viability and transcriptomic profiling of the two functionally selected CR-POPF effluents and were not complemented by protein-level cytokine measurements or downstream functional assays and therefore should not be interpreted as direct evidence of impaired healing-related cellular functions.

### Effluents representing biological complexity

By integrating metabolomics, viability assays, and transcriptomic profiling we suggest that the analyzed CR-POPF effluents represent compositionally diverse lipid environments rather than uniform pathological fluids. Reduced viability was confined to specific samples, indicating that biological activity depends on effluent composition rather than fistula classification alone. Metabolomic analysis revealed enrichment of long-chain saturated fatty acids (palmitic and stearic acid) and monoacylglycerides such as monopalmitin in CR-POPF effluents, consistent with intraperitoneal lipolysis models described in pancreatitis^10,17,18^. While we cannot fully disentangle in vivo from ex vivo contributions to lipid generation within the drain fluid, the observed lipid profiles showed reproducible patterns across patients and were associated with consistent functional effects in cellular assays, supporting a biologically active rather than purely degradative lipid environment. These lipid species contributed to the principal separation between CR-POPF and control effluents and may be associated with a mechanistic link between postoperative lipase leakage and exposure to cellular compartments relevant to anastomotic healing. Functional screening demonstrated differential ATP-based metabolic responses across effluents. Within sublethal exposure ranges (0.2 mM reference threshold), most effluents induced limited functional impairment, whereas two CR-POPF samples (AES1448 and GR1479) reproducibly reduced cellular viability. Importantly, these bioactive samples were characterized by higher lipid burden in the metabolomic screen, supporting a quantitative relationship between lipid concentration and effect magnitude. Together, these findings support a model in which the selected postoperative effluents act as complex biochemical mixtures capable of eliciting graded, compartment-specific adaptation rather than uniformly inducing cytotoxic injury. Nevertheless, these findings should be interpreted as mechanistic exemplars derived from functionally selected effluents rather than as a comprehensive representation of CR-POPF biology. In addition, elevated drain amylase levels, used clinically as a surrogate of pancreatic enzyme activity, were consistently associated with CR-POPF in our cohort, further supporting ongoing enzymatic activity as a biologically relevant component of the effluent environment.

### Defined lipolysis-derived reference stimulus

To experimentally anchor the observed lipid-responsive response, monopalmitin was used as a chemically defined lipolysis-derived reference stimulus rather than as a separate mechanistic entity. As a physiologically plausible monoacylglyceride generated during intraperitoneal triglyceride breakdown under pancreatic lipase exposure, it provides a controllable surrogate for lipid-rich effluent exposure^19,20^. The applied concentration (0.2 mM) represents a standardized sublethal reference condition rather than a direct approximation of in vivo concentrations, which cannot be reliably inferred from the available metabolomic profiling data. Monopalmitin did not induce a qualitatively distinct response but reproduced the same core pattern observed under selected CR-POPF effluent exposure at higher quantitative amplitude. This positions monopalmitin as an upper-bound reference within a graded model of lipid-driven activation in mesothelial cells and fibroblasts.

### Shared lipid-centered transcriptional framework

Using integrated metabolomic and transcriptomic profiling, we observed a coordinated lipid-responsive transcriptional signal linking effluent composition to structured cellular adaptation of mesothelial cells and fibroblasts. Transcriptional activation was not uniform across samples, supporting the concept that distinct lipid constellations are associated with differential biological activity. Thus, transcriptional responses induced by pancreatic fistula effluents partially overlap with the lipid-driven program, while additional variability likely reflects effluent-specific biochemical composition. This observation aligns with experimental evidence implicating intraperitoneal lipolysis as a potential contributor to postoperative inflammation and fistula progression^22^.

At the gene level, ANGPTL4 emerged as the most consistently induced transcript across stimuli and cell types. Together with coordinated upregulation of lipid-handling and metabolic regulators including PLIN2, PDK4, CPT1A, ACAA2, and OLAH, this pattern indicates structured metabolic reprogramming rather than isolated transcriptional events. Stress-associated transcripts such as HMOX1, STEAP1, and FKBP5 were induced in parallel, linking lipid handling to redox adaptation. Under higher-amplitude stimulation, additional chemokine-associated transcripts including CXCL8 became more prominent, suggesting broader transcriptional dispersion at increased lipid burden. Collectively, these findings were consistent with a graded spectrum of lipid-associated stress intensity rather than distinct mechanistic programs. This coordinated gene-level program was paralleled at the pathway level.

Across all conditions, pathway-level integration revealed a common lipid-centered configuration dominated by metabolic programs (HALLMARK_HYPOXIA, ADIPOGENESIS, GLYCOLYSIS; REACTOME_METABOLISM_OF_LIPIDS, REGULATION_OF_LIPID_METABOLISM_BY_PPARALPHA) together with graded engagement of stress-associated modules. Higher lipid burden—most prominently under monopalmitin exposure—amplified TNFα/NF-κB, mTORC1, and unfolded protein response pathways, supporting a quantitative stress-threshold model rather than stimulus-specific divergence. Taking together, these convergent gene- and pathway-level observations support the presence of a shared lipid-associated transcriptional framework within the studied conditions, that may be quantitatively modulated by lipid exposure across peritoneal mesothelial cells and fibroblasts.

### Cell-type–specific amplification

The in vitro system incorporated primary human peritoneal mesothelial cells together with fibroblasts as representative cellular compartments of the postoperative peritoneal environment relevant to anastomotic healing. By focusing on non-epithelial compartments and excluding tumor-derived cell lines, this approach enables interrogation of early cell responses in a controlled setting. We acknowledge that HFF do not capture the heterogeneity of pancreas-derived fibroblasts or pancreatic stellate cells. Accordingly, fibroblast findings should be interpreted as responses of a standardized stromal reference model rather than as definitive evidence for pancreas-specific stromal compartments.

Although a shared lipid-responsive transcriptional framework was preserved, response magnitude and contextual embedding varied according to cellular origin. Compartment-specific amplification was evident at both the gene and pathway level. While the metabolic core remained in common, its amplitude and downstream embedding differed between cell types. Peritoneal mesothelial cells exhibited broader transcriptional shifts, with stronger induction of stress-associated transcripts and enhanced expression of chemokine-related genes under higher lipid burden. Fibroblasts, by contrast, demonstrated a comparatively restricted response largely confined to lipid-handling and proteostasis-related programs.

These distinctions were reinforced at the pathway level. Mesothelial cells showed broader convergence with inflammatory and cytokine-proximal modules, including stronger overlap with innate immune activation signatures from the MSigDB C7 immunologic signature collection and immune-like reference profiles from the MSigDB C8 cell type signature collection without loss of lineage identity. Fibroblasts retained comparatively stronger enrichment of lipid-processing and proteostatic programs, including lipase complex assembly pathways (LPL/LIPC), with more constrained immune embedding. External anchoring through signatures derived from the MSigDB C2-CGP (curated chemical and genetic perturbation) collection further supported convergence with experimentally defined oxidative and metabolic stress states.

Overall, lipid-associated exposure engages a shared cellular response in peritoneal mesothelial cells and fibroblasts that is quantitatively amplified and differentially embedded depending on cellular origin, rather than giving rise to fundamentally distinct mechanistic pathways. Importantly, key transcriptional responses, including induction of ANGPTL4 and PDK4, were consistently observed in primary human peritoneal mesothelial cell preparations, supporting the physiological relevance of the identified lipid-responsive program.

### Translational and future perspectives

The present results delineate the transcriptional architecture, rather than a complete mechanistic signaling pathway. The data are derived from transcriptome profiling and integration at the signaling pathway level and therefore require independent validation at the proteome and functional levels before definitive mechanistic conclusions can be drawn.

In particular, cytokine secretion assays such as CXCL8/IL-8 ELISA, stromal migration or wound-healing assays, and validation in pancreas-derived fibroblasts or pancreatic stellate cells would be required to establish downstream functional relevance.

Nevertheless, rather than indicating stimulus-specific mechanisms, our data reveal a consistent lipid-responsive transcriptional signal derived from functionally selected CR-POPF effluents whose magnitude scales with lipid burden and whose embedding differs by cellular origin. Given the limited sample size, these findings should be interpreted as hypothesis-generating rather than generalizable. Increasing lipid exposure intensifies stress- and immune-associated signaling within this shared framework, rather than triggering a distinct cytotoxic program. Within this framework, the peritoneal mesothelium serves as a lipid-responsive interface of the peritoneal cavity, integrating lipid-associated exposure into alarm- and barrier stress–associated transcriptional programs. In contrast, fibroblasts exhibit a more metabolically centered adaptation with comparatively limited immune embedding, consistent with a role in matrix stabilization rather than alarm amplification.

In the context of CR-POPF, this compartment-specific integration may represent one potential mechanism influencing early barrier dynamics, local cytokine milieus, and coordination of repair processes. Clinically, this graded and cell type–dependent stress integration may provide a plausible explanation for the heterogeneous biological trajectories observed in CR-POPF, where differences in local lipid load may translate into variable activation and disease severity of mesothelial cells and fibroblasts. Thus, clinical severity may in part reflect the amplitude of lipid-dependent activation at the peritoneal interface. While the observed lipid signatures raise the possibility of their use as early biomarkers, such applications require prospective validation and cannot be inferred from the present data. Beyond biomarker development, the identified lipid-dependent response framework may also point toward potential therapeutic strategies, such as modulation of postoperative lipolytic activity, which warrants further investigation. Notably, randomized data indicate that purely technical reinforcement strategies have not consistently reduced clinically relevant POPF^21^. Conversely, interventions aimed at modifying the local biological environment, such as omental wrapping of the pancreaticojejunostomy, have been associated with reduced CR-POPF rates in recent meta- analyses^22^. In parallel, meta-analytic data demonstrate that intra-pancreatic fat deposition is associated with increased risk of clinically relevant POPF after pancreatoduodenectomy, further underscoring the relevance of tissue biology beyond purely technical factors^23^.

## Conclusion

These findings suggest that cellular compartments at the anastomotic interface may contribute to the biological response to lipid-rich CR-POPF effluents. The functionally selected lipid-rich CR-POPF effluents analyzed in this study were associated with shared but quantitatively graded transcriptional signals, with different amplitudes and embeddings between peritoneal mesothelial cells and fibroblasts. This is consistent with the concept that clinically relevant POPF may involve lipid-dependent stress integration in cellular compartments relevant to anastomotic healing, in addition to mechanical vulnerability. Future studies should validate these transcriptional signatures at the proteomic and functional levels, including effects on matrix remodeling, cytokine signaling, and barrier integrity. Integrating effluent composition, architecture of cellular response in peritoneal mesothelial cells and fibroblasts, and clinical parameters may guide perioperative strategies beyond purely technical optimization.

### Limitations

The study has limitations. The number of CR-POPF effluents was limited, and one sample was excluded due to technical failure, reducing statistical power. Transcriptomic analyses were restricted to two functionally active CR-POPF effluents, introducing a functional selection bias that limits generalizability. The in vitro design precludes causal inference and lacks in vivo validation. Early transcriptional response in peritoneal mesothelial cells and fibroblasts were isolated by excluding PDAC cell lines, focusing the analysis on mesothelial and cellular compartments relevant to anastomotic healing, but limiting direct comparability to tumor-adjacent tissue. Primary human foreskin fibroblasts served as a standardized stromal reference model relevant to anastomotic healing; this improves experimental control, but does not capture pancreas-derived fibroblast heterogeneity, pancreatic stellate cell biology, or pre-activated inflammatory states. Analyses were restricted to the transcriptomic level without proteomic validation or assessment of post-transcriptional regulation. In addition, the origin of lipid species detected in drain effluents cannot be definitively attributed to in vivo processes, as contributions from ex vivo enzymatic activity within the drain fluid cannot be excluded. Furthermore, functional readouts were limited to ATP-based metabolic viability assays as a surrogate of lipid-induced cellular stress responses and did not include matrix remodeling, cytokine secretion, barrier integrity, or repair-associated endpoints. Consequently, the findings describe pathway-level transcriptional architectures rather than direct functional consequences. Finally, pooled transcriptomic analyses were used to evaluate shared pathway patterns. Concordance with individual sample-level analyses indicates internal consistency of the observed patterns within the analyzed conditions within the analyzed functionally selected conditions.

## Materials and methods

### Data collection and patient samples

Clinical data were derived from a continuously maintained, institution-specific database of the Department of General and Visceral Surgery at the University Medical Center Freiburg. This database prospectively records patient data at the time of treatment as part of routine clinical practice and is approved by the local ethics committee. All patients provided written informed consent for the use of their clinical data for research purposes. For the purposes of this study, these data were analyzed retrospectively. Patients who underwent pancreatic head resection for suspected PDAC between 2019 and 2025 were included. Postoperative abdominal drainage effluents were collected on the third postoperative day, prior to the clinical manifestation of CR-POPF. Patients were subsequently classified retrospectively into CR-POPF (*n* = 7) and non-POPF (*n* = 7) groups according to ISGPS criteria.

All experimental analyses, including metabolomic profiling of drain effluents and cell culture experiments, were performed specifically within the scope of this study. RNA isolation and transcriptomic profiling were newly conducted, and sequencing data were generated specifically for the present project.

The study was conducted in accordance with the Declaration of Helsinki and approved by the Ethics Committee of the University of Freiburg (ID: 23-1302-S1). The study adheres to the MDAR framework and its sequencing data comply with MINSEQE standards^24,25^.

### Surgical procedure of pancreaticoduodenectomy

At our high-volume center, experienced pancreatic surgeons performed all pancreatic head resections as standard Whipple, pylorus-resecting or pylorus-preserving pancreaticoduodenectomy. In all cases analyzed, reconstruction consisted of duct-to-mucosa pancreatojejunostomy, hepaticojejunostomy and duodeno/ gastrojejunostomy. The jejunal loop was positioned in Child-S or Roux-en-Y orientation. One or two drains were routinely placed adjacent to the pancreatic anastomosis^26^.

### Inclusion/exclusion criteria

Eligible patients were adults (≥ 18 years old) who underwent pancreatic head resection for suspected pancreatic ductal adenocarcinoma (PDAC) and had complete clinical, operative, and pathological data, as well as available postoperative drainage fluid within 72 h after surgery. Patients were excluded if they underwent pancreatic resection for reasons other than PDAC, had a history of pancreatic surgery, had an intra-abdominal infection or sepsis at the time of resection, or had insufficient or contaminated drainage fluid samples (less than 2 mL, hemolysis, or bacterial overgrowth). Patients lost to follow-up within 30 days were also excluded.

### Patient consent

All patients were informed prior to surgery about the potential use of their biological samples and associated clinical data for research purposes and provided written informed consent. Samples were collected and stored within the institutional Organoid/Freeze Biobank at the University Medical Center Freiburg. Data were stored in a pseudonymized form according to institutional and national regulations.

### Sex and gender reporting (SAGER guidelines)

This study was conducted in accordance with the Sex and Gender Equity in Research (SAGER) guidelines. Primary human peritoneal mesothelial cell preparations were derived from pseudonymized donors. Information on biological sex was not available to the investigators for these primary cell preparations, precluding sex-disaggregated analyses in the cell-based experiments. PanC-1 cells and human foreskin fibroblasts (HFF) were obtained from commercial sources. No sex-specific analyses were performed in these established in vitro models, and the experimental design did not incorporate sex as a biological variable. For pancreatic effluent samples, donor sex was recorded and is reported in Table 1. Sex-related information was therefore disclosed for all human-derived materials for which such data were available.

### Statistical analysis

Clinical and perioperative data were analyzed using SPSS (IBM SPSS Statistics, version 30; IBM Corp., Armonk, NY, USA) and in R (version 4.5.0; R Core Team). Continuous variables are reported as median (interquartile range) or mean ± SEM, as appropriate. Group comparisons between patients with and without CR-POPF were performed using Student’s t test or Mann–Whitney U test for continuous variables and χ² test or Fisher’s exact test for categorical variables. A two-sided p value < 0.05 was considered statistically significant. For tables with more than two categorical levels, Pearson’s chi-square test (two-sided) was applied, whereas Fisher’s exact test was used for 2 × 2 tables. For in vitro assays, viability and cytotoxicity data were analyzed using two-way ANOVA with time and treatment as fixed factors. Where appropriate, repeated measures two-way ANOVA on normalized (% of control) values were used to account for within-experiment correlations. Post hoc comparisons were adjusted for multiple testing as indicated in the figure legends.

### Metabolomic analysis

500 µl of pancreas drainage fluid was extracted by adding 500 µl chloroform containing 2 µg/ml methyl nonadecanoate as internal standard. After shaking for 5 min at 1400 rpm, samples were centrifuged for 10 min at 20,000 g and 4 °C. 200 µl of the lower phase were transferred into a new reaction tube and evaporated in a vacuum concentrator. Dried lipids were derivatized by adding 20 µl pyridine and 50 µl N-methyl-N-trimethylsilyl trifluoroacetamide and incubated for 30 min at 37 °C and 1200 rpm. After 2 min centrifugation at 20,000 g at room temperature, 30 µl were transferred into a GC/MS vial and 30 µl of each sample were used to prepare a pooled quality control sample. Samples were injected in randomized order with regularly injected quality control samples in between using a GC-EI-MS equipped with an HP5-MS column as previously described^27^. Intensities of each lipid were normalized to the internal standard and analyzed using the MetaboAnalyst 6.0^28^. Palmitic acid, monopalmitin, and stearic acid were selected for functional testing based on their enrichment profiles in the metabolomic screen. Monopalmitin arises from partial lipolysis of triglycerides and therefore reflects active lipase-mediated fat degradation rather than passive release of free fatty acids. Because of its higher aqueous stability and solubility, monopalmitin allowed reproducible dose–response testing in cell culture and was thus used as the primary stimulus for transcriptomic experiments. A total of 24 drain effluent samples (12 CR-POPF and 12 controls) were initially subjected to metabolomic profiling. For downstream functional and transcriptomic experiments, a subset of 14 samples (7 CR-POPF and 7 controls) was selected according to predefined technical criteria, including sample integrity and sufficient residual volume for standardized in vitro exposure experiments. Samples not included in downstream analyses were excluded because of insufficient remaining volume or preanalytical limitations. Excluded samples did not differ in baseline clinical characteristics or in overall lipid enrichment patterns.

### Cell lines and culture conditions

#### Human foreskin fibroblasts (HFF)

Human foreskin fibroblasts (HFF; ATCC^®^ SCRC-1041™) were obtained from the American Type Culture Collection. Cells were cultured in Dulbecco’s Modified Eagle Medium (DMEM, high glucose, GlutaMAX) supplemented with 10% heat-inactivated fetal bovine serum (FBS, lot-matched), 1% penicillin–streptomycin, and 2 mmol/L L-glutamine at 37 °C in a humidified incubator with 5% CO₂. HFF were used for experiments at passages 6–10 and routinely tested negative for mycoplasma contamination.

#### Peritoneal mesothelial cells

Peritoneal mesothelial cells were isolated from omental tissue obtained from randomly selected non-oncological resection specimens preserved in the institutional Organoid/Freeze Biobank at the University Medical Center Freiburg between 2020 and 2022. Only samples derived from the omentum majus were used for this study. Tissue collection and storage followed standardized biobanking protocols. Cells were isolated by enzymatic digestion and expanded in appropriate growth medium. Experiments were performed using five independent peritoneal mesothelial cell preparations at passages 3–7.

#### Pancreatic carcinoma cells (PanC-1 cell line)

Pancreatic carcinoma cells (PanC-1 cell line; ATCC^®^ CRL-1469™), originally derived from a human pancreatic ductal adenocarcinoma (PDAC), were used as a tumor-derived epithelial cell line. Cells were maintained in DMEM and used at passages below 15. PanC-1 cells were excluded from transcriptomic analyses to avoid confounding effects of tumor-intrinsic genomic and transcriptional alterations and to focus on cell–specific responses relevant to anastomotic healing.

### Assays for cell viability and cytotoxicity

#### Luminescent cell viability assay (CellTiter-Glo^®^)

Cell viability was assessed using the CellTiter-Glo^®^ Luminescent Cell Viability Assay (Promega, Madison, WI, USA), according to the manufacturer’s instructions. CellTiter-Glo quantifies intracellular ATP as a surrogate marker of metabolically active cells. Upon addition of the reagent containing luciferase and luciferin, cells are lysed and generate a stable luminescent signal (half-life > 5 h). Light intensity is directly proportional to the number of viable cells.

#### Fatty acid challenge across peritoneal mesothelial cells, pancreatic cells and fibroblasts

HFF, PanC-1, and peritoneal mesothelial cell preparations were subjected to a series of dose–response experiments to assess cellular tolerance to individual lipid composition. Cells were cultured in serum-free medium supplemented with fatty acid–poor bovine serum albumin (BSA) as a carrier. Treatments included vehicle control (ethanol) and three lipid species, palmitic acid (PA), stearic acid (SA), and monopalmitin (MP), each complexed to BSA. Concentrations of 0.1 mM, 0.2 mM, and 0.5 mM were applied for 24 h under standard culture conditions (37 °C, 5% CO₂). After incubation, cell viability and cytotoxicity were assessed using standardized metabolic and membrane integrity–based assays. Data were summarized as mean ± SEM, and statistical testing was performed as indicated in the figure legends.

#### Exposure to postoperative effluents

In the second experimental step, cell viability and cytotoxicity assays were conducted using postoperative drain effluents obtained from patients with and without CR-POPF. Two experimental setups were established using non-fistula effluents and CR-POPF effluents, respectively. PanC-1, HFF, and peritoneal mesothelial cell preparations were exposed to patient-derived effluents diluted 1:5 (effluent: medium) in serum-free culture medium supplemented with fatty acid–poor bovine serum albumin (BSA). After equilibration, the culture medium was replaced with effluent-supplemented medium. Effluents from seven patients per group (non-fistula and CR-POPF) were used. For each condition, three biological replicates were performed per cell line. Cells were incubated with the respective effluents for 24, 48, or 72 h. After each incubation period, cell viability and cytotoxicity were assessed using standardized metabolic and membrane integrity–based assays, and mean values per cell line were recorded.

#### RNA isolation and sequencing for transcriptomic profiling

Peritoneal mesothelial cell preparations were initially available from six independent non-malignant donors (P1–P6). One preparation (P2) was excluded from downstream transcriptomic analysis because of technical failure during laboratory processing. Consequently, RNA-seq analyses were performed on five evaluable mesothelial preparations. Human foreskin fibroblasts (HFF) were used as a reference fibroblast cell line. Both cell types were exposed for 24 h to postoperative effluents selected based on prior viability screening. Effluents from two CR-POPF patients (AES1448 and GR1479) were used, alongside medium alone (CTRL) and monopalmitin (0.2 mM) as controls. For transcriptomic profiling, HFFs and peritoneal mesothelial cell preparations were re-exposed to the selected effluents diluted 1:50 (effluent: medium) in serum-free culture medium to ensure sufficient cell viability for RNA isolation. For each cell type (peritoneal cells and HFF) and condition (CTRL, AES1448, GR1479, monopalmitin 0.2 mM), three independent culture replicates were prepared. After exposure, total RNA was isolated using silica-membrane spin columns (RNeasy Mini Kit, Qiagen, Hilden, Germany) with on-column DNase digestion. RNA concentration and purity were determined spectrophotometrically, and integrity was verified by microcapillary electrophoresis; only samples with RIN ≥ 8 were used for sequencing. RNA sequencing libraries were prepared by Novogene GmbH (Planegg, Germany) using the TruSeq Stranded mRNA Library Prep Kit (Illumina) and sequenced on a NovaSeq 6000 platform (2 × 100 bp; ~25–30 million reads per sample). Raw reads underwent quality control using FastQC and MultiQC, adapter trimming with Cutadapt, alignment to the human reference genome (GRCh38) using STAR, and gene-level read quantification with featureCounts. Differential expression analysis was performed on raw count data using DESeq2, applying a threshold of |log₂ fold change| ≥ 0.5 and a Benjamini–Hochberg–adjusted false discovery rate (FDR) < 0.05.

#### Transcriptomics analysis

Paired-end reads were trimmed to remove low-quality bases and adapter sequences using Trimmomatic (v0.39). edgeR was used for complementary differential expression and enrichment analyses to ensure robustness across normalization and statistical frameworks. Trimmed reads were aligned to the human reference genome (hg38), and reads per gene were quantified using STAR (v2.7.11a). Downstream analysis was performed in R (v4.4.0). Lowly expressed genes were removed using the filterByExpr function from the edgeR package, which retains only those genes with sufficient read counts (minimum of 10 counts in at least 70% of samples within a group, and a total count of at least 15 across all samples) to ensure reliable statistical inference. Counts were normalized by library size and the trimmed mean of M-values (TMM) method. Principal component analysis (PCA) of TMM-normalized log₂ counts per million (CPM) was performed to explore sample variance and clustering. Differential gene expression analysis was analyzed (i) between treatments within the same patient and (ii) across patients and healthy donor controls, with the Bioconductor package edgeR (v4.2.2). Genes were considered differentially expressed at |log₂ fold change| ≥ 0.5 and false discovery rate (FDR) < 0.05. Gene-set enrichment was performed using clusterProfiler (v4.12.6) with MSigDB as reference gene-sets. Enrichment scores were normalized (NES) and adjusted for multiple testing (FDR < 0.05). Heatmaps were generated to visualize expression patterns of top-ranked differentially expressed genes and enriched pathways. Keyword-based searches (“lipo”, “toxi”) were applied to enrichment outputs to highlight mechanistically relevant processes. To integrate results across conditions, Venn diagrams were constructed to illustrate overlaps between DEG sets, and significance of intersections was evaluated by Fisher’s exact test using the union of all differentially expressed genes as the reference universe. Supplementary materials include volcano plots, representative DEG lists, and detailed enrichment results corresponding to the main analyses.

A graphical overview summarizing the study design, experimental workflow and analytical strategy is provided in Supplementary Graphical Abstract.

## Supplementary Information

Below is the link to the electronic supplementary material.

Supplementary Material 1

## Data Availability

Underlying metabolomic and transcriptomic analyses as well as patient-level data not included in the Supplementary Materials are available from the corresponding author upon reasonable request. RNA-seq data have been deposited in the Gene Expression Omnibus (GEO) under accession number GSE319229 and are accessible at [https://www.ncbi.nlm.nih.gov/geo/query/acc.cgi?acc=GSE319229](https:/www.ncbi.nlm.nih.gov/geo/query/acc.cgi? acc=GSE319229) . Reviewer access is provided via the following token: afshgskqpfirzad.

## Abbreviations

AES1448: CR–POPF effluent sample (patient code)
ANOVA: Analysis of variance
BSA: Bovine serum albumin
CR: POPF–Clinically relevant postoperative pancreatic fistula
DEGs: Differentially expressed genes
DMEM: Dulbecco’s modified eagle medium
FBS: Fetal bovine serum
FDR: False discovery rate
GC: MS–Gas chromatography–mass spectrometry
GSEA: Gene set enrichment analysis
GR1479: CR–POPF effluent sample (patient code)
HFF: Human foreskin fibroblasts
ISGPS: International study group on pancreatic surgery
MDAR: Materials, design, analysis and reporting
MINSEQE: Minimum Information about a high–throughput sequencing experiment
MP: Monopalmitin
NES: Normalized enrichment score
NF: κB–Nuclear factor kappa–light–chain–enhancer of activated B cells
PA: Palmitic acid
PanC: Pancreatic carcinoma cells (PanC–1 line)
PCA: Principal component analysis
PDAC: Pancreatic ductal adenocarcinoma
POPF: Postoperative pancreatic fistula
QC: Quality control
RNA: seq–RNA sequencing
SA: Stearic acid
SPSS: Statistical Package for the Social Sciences
UPR: Unfolded protein response

## References

[1] Chui, J. N., Sahni, S., Samra, J. S. & Mittal, A. Postoperative pancreatitis and pancreatic fistulae: a review of current evidence. *HPB* **25** (9), 1011–1021. 10.1016/j.hpb.2023.05.007 (2023). 37301633 10.1016/j.hpb.2023.05.007

[2] Marchegiani, G. & Bassi, C. Prevention, prediction, and mitigation of postoperative pancreatic fistula. *Br. J. Surg.* **108** (6), 602–604. 10.1093/bjs/znab125 (2021). 33942063 10.1093/bjs/znab125

[3] Cameron, J. L., Riall, T. S., Coleman, J. & Belcher, K. A. One thousand consecutive pancreaticoduodenectomies. *Ann. Surg.* **244** (1), 10–15. 10.1097/01.sla.0000217673.04165 (2006). .ea PubMed PMID: 16794383; PubMed Central PMCID: PMC1570590. 16794383 10.1097/01.sla.0000217673.04165.ea PMC1570590

[4] Theijse, R. T. et al. Nationwide Outcome after Pancreatoduodenectomy in Patients at Very High Risk (ISGPS-D) for Postoperative Pancreatic Fistula. *Ann. Surg.* **281** (2), 322. 10.1097/SLA.0000000000006174 (2025). 10.1097/SLA.0000000000006174 PMC11723487 38073575

[5] Bassi, C. et al. The 2016 update of the International Study Group (ISGPS) definition and grading of postoperative pancreatic fistula: 11 Years After. *Surgery* **161** (3), 3. 10.1016/j.surg.2016.11.014 (2017). PubMed PMID: 28040257. 10.1016/j.surg.2016.11.014 28040257

[6] Khalid, A. et al. Impact of postoperative pancreatic fistula on outcomes in pancreatoduodenectomy: a comprehensive analysis of American College of Surgeons National Surgical Quality Improvement Program data. *J. Gastrointest. Surg.* **28** (9), 1406–1411. 10.1016/j.gassur.2024.05 (2024). .035 PubMed PMID: 38821210. 38821210 10.1016/j.gassur.2024.05.035

[7] Eshmuminov, D. et al. Systematic review and meta-analysis of postoperative pancreatic fistula rates using the updated 2016 International Study Group Pancreatic Fistula definition in patients undergoing pancreatic resection with soft and hard pancreatic texture. *HPB (Oxford)*. **20** (11), 992–1003. 10.1016/j.hpb.2018.04.003 (2018). PubMed PMID: 29807807. 29807807 10.1016/j.hpb.2018.04.003

[8] Uchida, Y. et al. Clinical and experimental studies of intraperitoneal lipolysis and the development of clinically relevant pancreatic fistula after pancreatic surgery. *Br. J. Surg.* **106** (5), 5. 10.1002/bjs.11075 (2019). PubMed PMID: 30725479. 10.1002/bjs.11075 30725479

[9] Nemecz, M. et al. The Distinct Effects of Palmitic and Oleic Acid on Pancreatic Beta Cell Function: The Elucidation of Associated Mechanisms and Effector Molecules. *Front. Pharmacol.* **9**10.3389/fphar.2018.01554 (2019). 10.3389/fphar.2018.01554 PMC6348268 30719005

[10] Durgampudi, C. et al. Acute Lipotoxicity Regulates Severity of Biliary Acute Pancreatitis without Affecting Its Initiation. *Am. J. Pathol.* **184** (6), 1773–1784. 10.1016/j.ajpath.2014.02.015 (2014). PubMed PMID: 24854864; PubMed Central PMCID: PMC4044711. 24854864 10.1016/j.ajpath.2014.02.015 PMC4044711

[11] Marchegiani, G. et al. Postpancreatectomy Acute Pancreatitis (PPAP): Definition and Grading From the International Study Group for Pancreatic Surgery (ISGPS). *Ann. Surg.* **275** (4), 663. 10.1097/SLA.0000000000005226 (2022). 34596077 10.1097/SLA.0000000000005226

[12] Vlăduț, C. et al. High prevalence of pancreatic steatosis in pancreatic cancer patients: A meta-analysis and systematic review. *Pancreatology* **25** (1), 98–107. 10.1016/j.pan (2025). .2024.11.010 PubMed PMID: 39706752. 39706752 10.1016/j.pan.2024.11.010

[13] Patel, K. et al. Lipolysis of Visceral Adipocyte Triglyceride by Pancreatic Lipases Converts Mild Acute Pancreatitis to Severe Pancreatitis Independent of Necrosis and Inflammation. *Am. J. Pathol.* **185** (3), 808–819 (2015). PubMed PMID: 25579844; PubMed Central PMCID: PMC4348470. 25579844 10.1016/j.ajpath.2014.11.019 PMC4348470

[14] Liu, Q. et al. Long-chain fatty acids - The turning point between mild and severe acute pancreatitis. *Heliyon* **10** (11), 11. 10.1016/j.heliyon.2024.e31296 (2024). PubMed PMID: 38828311; PubMed Central PMCID: PMC11140623. 10.1016/j.heliyon.2024.e31296 PMC11140623 38828311

[15] Xia, W., Lu, Z., Chen, W., Zhou, J. & Zhao, Y. Excess fatty acids induce pancreatic acinar cell pyroptosis through macrophage M1 polarization. *BMC Gastroenterol.* **22** (1), 72. 10.1186/s12876-022-02146-8 (2022). 35183119 10.1186/s12876-022-02146-8 PMC8858517

[16] Leishman, S. et al. Fatty acid synthesis promotes inflammasome activation through NLRP3 palmitoylation. *Cell. Rep.* **43** (8), 114516. 10.1016/j.celrep.2024.114516 (2024). PubMed PMID: 39024103. 39024103 10.1016/j.celrep.2024.114516

[17] Lilly, A. C., Astsaturov, I. & Golemis, E. A. Intrapancreatic fat, pancreatitis, and pancreatic cancer. *Cell. Mol. Life Sci.* **80** (8), 206. 10.1007/s00018-023-04855-z (2023). 37452870 10.1007/s00018-023-04855-z PMC10349727

[18] Okumura, T. et al. Extra-pancreatic invasion induces lipolytic and fibrotic changes in the adipose microenvironment, with released fatty acids enhancing the invasiveness of pancreatic cancer cells. *Oncotarget* **8** (11), 18280–18295. 10.18632/oncotarget.15430 (2017). PubMed PMID: 28407685; PubMed Central PMCID: PMC5392327. 28407685 10.18632/oncotarget.15430 PMC5392327

[19] Reis, P. et al. Competition between Lipases and Monoglycerides at Interfaces. *Langmuir* **24** (14), 7400–7407. 10.1021/la800531y (2008). 18547084 10.1021/la800531y

[20] Stork, E. et al. Enzymatic hydrolysis during *in vitro* digestion of triacylglycerols in a model system and milk: Isomerisation of diacylglycerols and monoacylglycerols. *Food Chem. Adv.* **8**, 101041. 10.1016/j.focha.2025.101041 (2025).

[21] Shibuya, K. et al. Effect of Double Layer Polyglycolic Acid Felt for Reducing Pancreatic Fistula After Pancreatoduodenectomy: Results of a Multicenter Randomized Control Trial (PLANET-PJ trial). *Ann. Surg.* **283** (3), 398–409 (2026). .0000000000006857 PubMed PMID: 40728222; PubMed Central PMCID: PMC12893145. 40728222 10.1097/SLA.0000000000006857 PMC12893145

[22] Mirza, W. et al. Omental wrapping of the pancreaticojejunostomy during pancreatoduodenectomy to prevent clinically relevant POPF: a GRADE-assessed systematic review and meta-analysis featuring geographic subgroup analysis. *World J. Surg. Oncol.* **24** (1), 54. 10.1186/s12957-026-04218-5 (2026). PubMed PMID: 41572283; PubMed Central PMCID: PMC12833943. 41572283 10.1186/s12957-026-04218-5 PMC12833943

[23] Waili, A. et al. Postoperative pancreatic fistula: a bibliometric analysis of research trends and a meta-analysis on the association with intra pancreatic fat deposition. *Sci. Rep.* **15** (1), 44360. 10.1038/s41598-025-27860-7 (2025). PubMed PMID: 41436493; PubMed Central PMCID: PMC12727792. 41436493 10.1038/s41598-025-27860-7 PMC12727792

[24] Brazma, A. et al. MINSEQE: Minimum Information about a high-throughput Nucleotide SeQuencing Experiment - a proposal for standards in functional genomic data reporting [Internet]. Jun **1**. 10.5281/zenodo.5706412 (2012).

[25] Chambers, K. et al. Towards minimum reporting standards for life scientists [Internet]. MetaArXiv; 2019 [cited 2025 Nov 8]. Available from: https://osf.io/9sm4x10.31222/osf.io/9sm4x

[26] Warren, K. W. & Cattell, R. B. Basic Techniques in Pancreatic Surgery. Surgical Clinics of North America. ;Symposium on Surgical Technique36(3):707–24. 10.1016/S0039-6109(16)34896-4 (1956). 10.1016/s0039-6109(16)34896-4 13324659

[27] Lagies, S. et al. Metabolic characterization of directly reprogrammed renal tubular epithelial cells (iRECs). *Sci. Rep.* **8** (1), 3878. 10.1038/s41598-018-22073-7 (2018). 29497074 10.1038/s41598-018-22073-7 PMC5832874

[28] Pang, Z. et al. MetaboAnalyst 6.0: towards a unified platform for metabolomics data processing, analysis and interpretation. *Nucleic Acids Res.* **52** (W1), W398–406. 10.1093/nar/gkae253 (2024). 38587201 10.1093/nar/gkae253 PMC11223798

