# Supplementary material for: Lipid signatures in two functionally selected effluents from clinically relevant postoperative pancreatic fistulas are associated with graded, cell type–specific transcriptional responses
Source: Sci Rep. 2026 Jul 23;16:23096. doi: 10.1038/s41598-026-63058-1 (PMC13396434; doi:10.1038/s41598-026-63058-1)
Supplement: Supplementary file 1 — Supplementary Material 1 [file 41598_2026_63058_MOESM1_ESM.docx]

**Lipid signatures in two functionally selected effluents from clinically relevant postoperative pancreatic fistulas are associated with graded, cell type–specific transcriptional responses**

Johannes D. Lettner^1*^, Marvin Schwarzer^1^, Simon Lagies^4,6^, Bernd Kammerer^3,4,5,6^, Stephanie Mewes^1^, Sophia Chikhladze^1^, Stefan Fichtner-Feigl^1^, Geoffroy Andrieux^7^, Dietrich A. Ruess^1,2,†^, Uwe A. Wittel^1,†^

**Supplemental Material**

1 Department of General and Visceral Surgery, Center for Surgery, Medical Center University of Freiburg, Faculty of Medicine, University of Freiburg, Freiburg, Germany

2 German Cancer Consortium (DKTK), Partner Site Freiburg and German Cancer Research Center (DKFZ), Heidelberg, Germany.

3 BIOSS Center of Biological Signaling Studies, University of Freiburg, Freiburg, Germany

4 Core Competence Metabolomics, Hilde-Mangold-Haus, University of Freiburg, Freiburg, Germany
5 Spemann Graduate School of Biology and Medicine (SGBM), University of Freiburg, Freiburg, Germany

6 Institute of Organic Chemistry, University of Freiburg, Freiburg, Germany

7 Institute of Medical Bioinformatics and Systems Medicine, Medical Center University of Freiburg, Faculty of Medicine, University of Freiburg, Freiburg, Germany

*Corresponding author, Johannes D. Lettner, Hugstetterstraße 55, 79106 Freiburg Germany, mail: [johannes.lettner@uniklinik-freiburg.de](mailto:johannes.lettner@uniklinik-freiburg.de), Tel.: +49 0761/270 23650

† Equal Contribution, shared last Authorship

**MDAR Reporting Checklist**

| **Domain** | **Item** | **Response** |
| --- | --- | --- |
| **Materials** | Newly created materials | No new materials generated; all analytes (lipids) commercially available. Not applicable. |
|  | Antibodies | Not applicable. No antibodies used. |
|  | DNA and RNA sequences | Not applicable. No novel sequences generated; sequencing performed on endogenous transcripts only. |
|  | Cell materials | Human fibroblast (HFF-1), peritoneal mesothelial cells, and pancreatic ductal epithelial cells (PanC-1) from certified repositories. Mycoplasma-negative. |
|  | Experimental animals | Not applicable. No in-vivo work performed. |
|  | Plants and microbes | Not applicable. |
|  | Human research participants | Drain effluents obtained from 14 surgical patients after informed consent; demographics and ethics approval reported in Methods. |
| **Design** | Study protocol | Mechanistic multi-omics study; not preregistered (exploratory translational design). |
|  | Laboratory protocol | Full protocols for GC-MS and RNA-seq provided in Methods; standard references cited. |
|  | Sample size determination | Not formally powered; exploratory study with n = 14 patient samples. |
|  | Randomization | Cell treatments randomized across plates. |
|  | Blinding | Investigators blinded to effluent group during viability assays and RNA-seq library preparation. |
|  | Inclusion/exclusion criteria | One effluent was excluded due to technical measurement failure; PDAC cell lines were deliberately excluded to avoid oncogenic bias. |
|  | Sample definition and replication | Viability and cytotoxicity assays were performed in triplicate (biological n = 3 per condition). RNA-seq analyses included three biological replicates per group (RIN ≥ 8; 25–30 M reads/sample). |
|  | Ethics | Approved by institutional ethics committee; informed consent obtained from all participants (Ethics ID: 23-1302-S1). |
|  | Dual Use Research of Concern (DURC) | Not applicable. |
| **Analysis** | Attrition | One effluent excluded due to technical measurement failure (2-way ANOVA dataset). |
|  | Statistics | Two-way ANOVA (stimulus × concentration) for viability/cytotoxicity; edgeR for differential expression (FDR < 0.05); GSEA for pathway enrichment. Detailed workup included in Methods. |
|  | Data normalization / QC | GC-MS data were normalized to internal standards and total ion count. RNA-seq data were processed as described in the RNA-seq statistical analysis section. |
|  | Data availability | RNA-seq data were processed and curated in accordance with ENCODE and MDAR standards. GC–MS data were generated and curated following the Metabolomics Standards Initiative (MSI) guidelines. RNA-seq data have been deposited in Gene Expression Omnibus (GSE319229). Additional processed data supporting the findings of this study are available from the corresponding author upon reasonable request and in accordance with institutional and ethical regulations. |
|  | Code availability | Custom R scripts supporting the analyses (R v4.4.0; edgeR, clusterProfiler) are available from the corresponding author upon reasonable request. |
| **Reporting** | Adherence to community standards | Study follows MDAR for transparency. MDAR checklist provided in Supplementary Information. |
|  |  |  |

**Graphical Abstract**


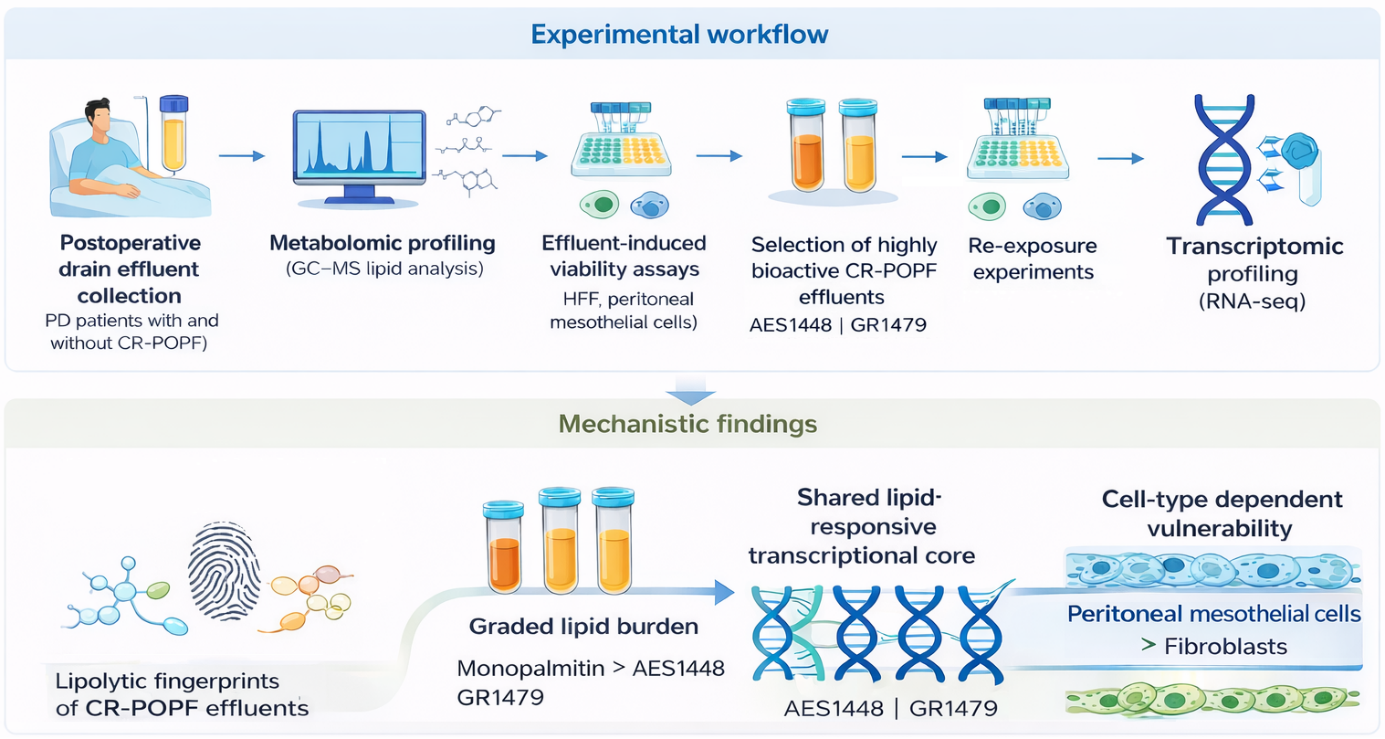


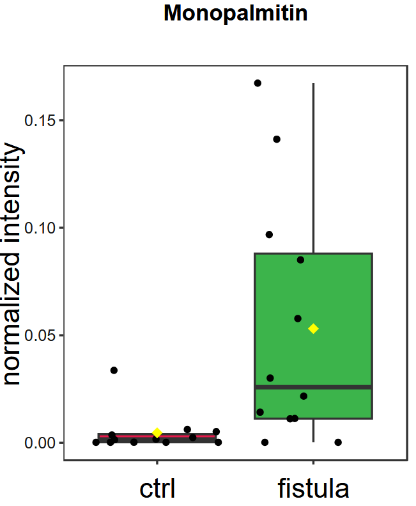
**Metabolomic and Transcriptomic Analysis**

**Figure S1. Box–swarm plot of monopalmitin levels.** Normalized monopalmitin intensities are shown for control and CR-POPF drain effluent samples. Boxes represent the median and interquartile range, with individual measurements overlaid. The between-group difference reached nominal statistical significance (unadjusted p < 0.05).


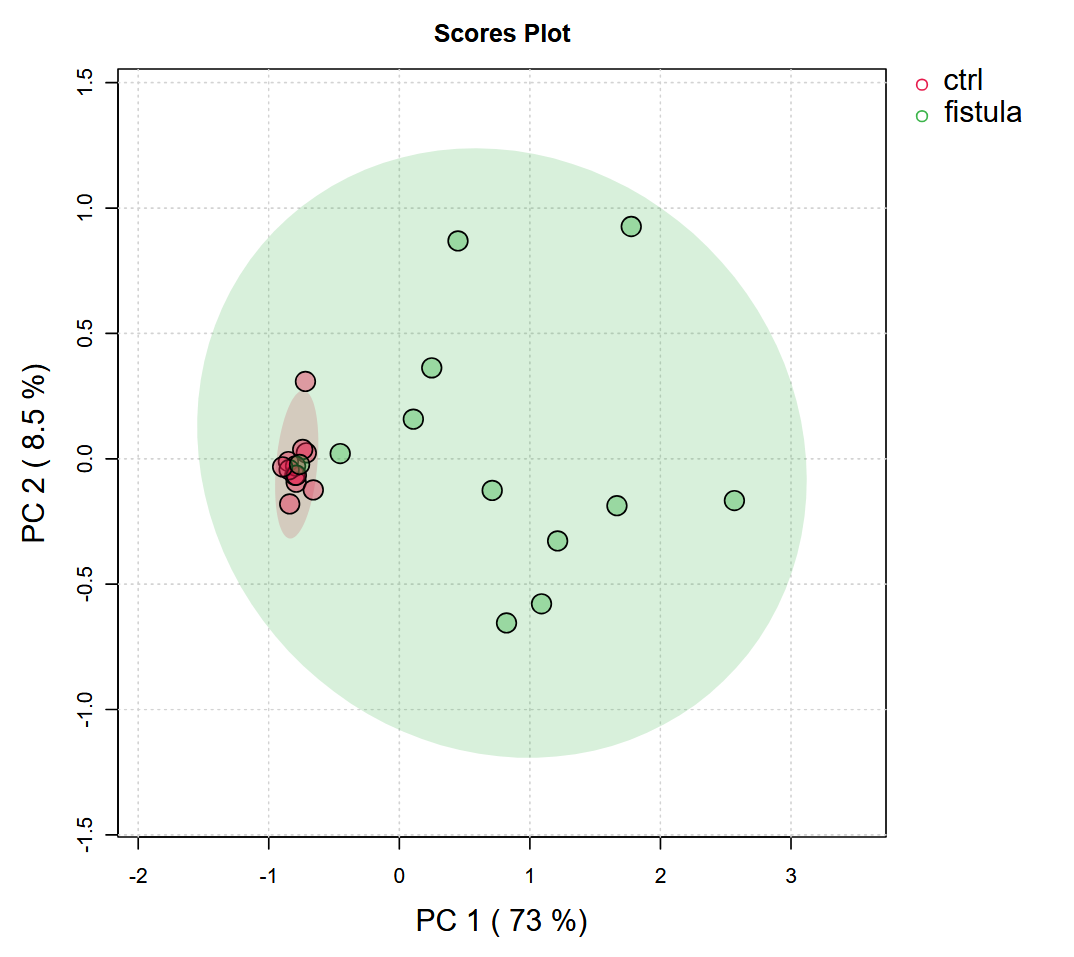


**Figure S2. Principal component analysis (PCA) of control and CR-POPF samples.** Unsupervised PCA scores plot showing the distribution of control and CR-POPF samples along the first two principal components (PC1 and PC2). Shaded ellipses represent 95% confidence regions for each group.


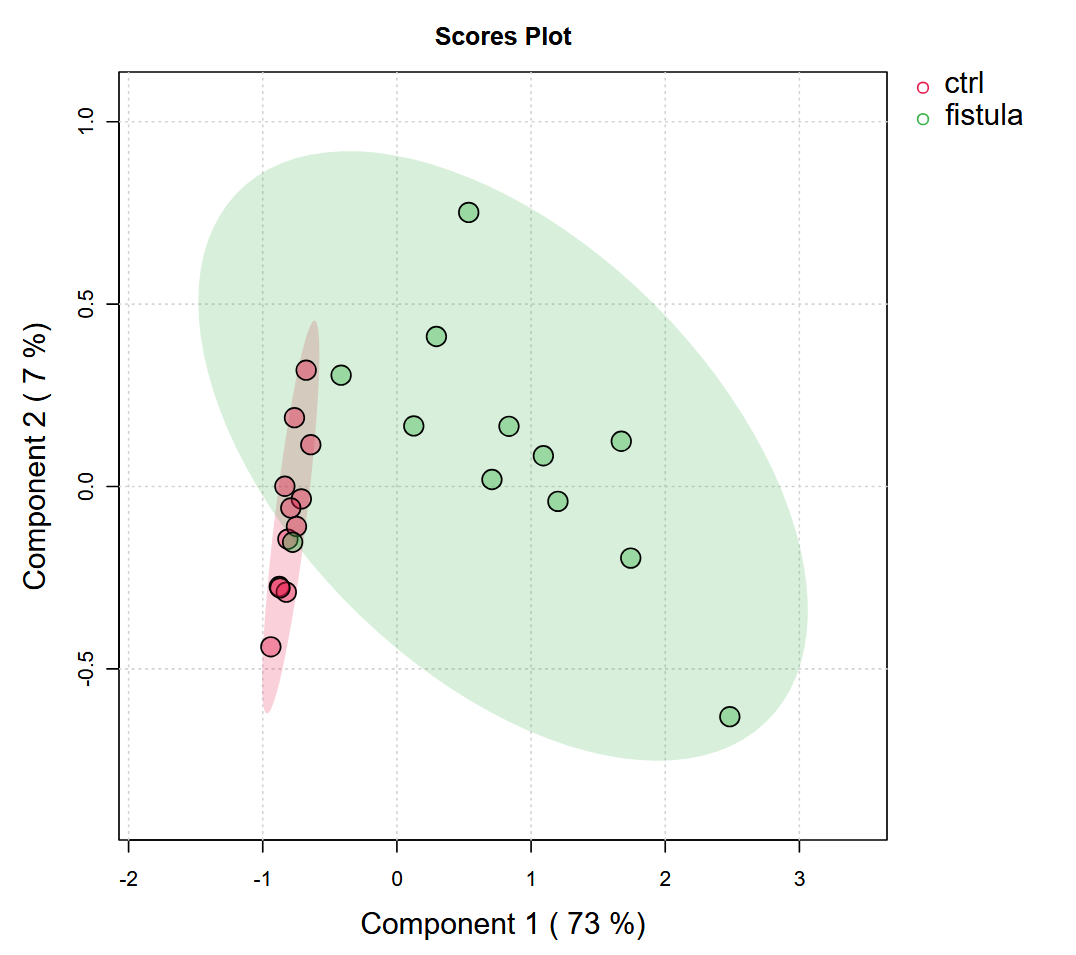


**Figure S3. Partial least squares discriminant analysis (PLS-DA) of control and CR-POPF samples.**PLS-DA scores plot showing the distribution of control and CR-POPF samples along the first two latent components. Shaded ellipses represent 95% confidence regions for each group.

**
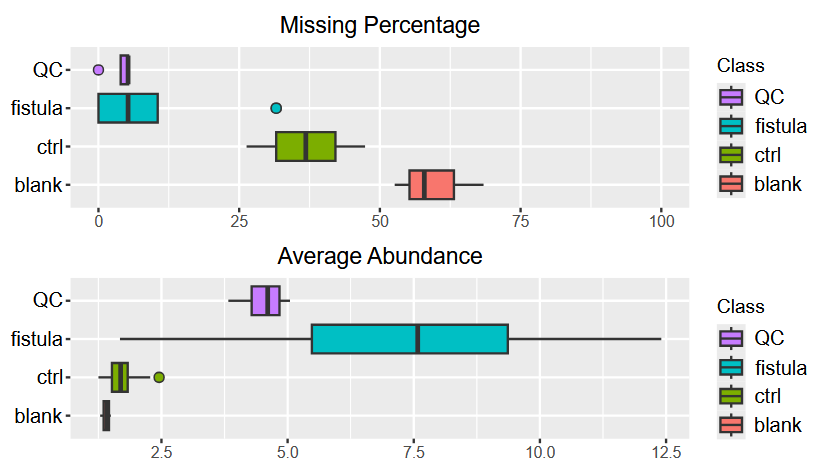
**

**Figure S4. Summary of the quality control metrics for the GC-MS analysis of postoperative drain effluents.** This figure shows the percentage of missing values across blank, control, CR-POPF, and pooled QC samples. The average signal abundance per class demonstrates a uniform instrument response. The stable intensity distributions of regularly injected pooled QC samples throughout the run confirm analytical reproducibility.


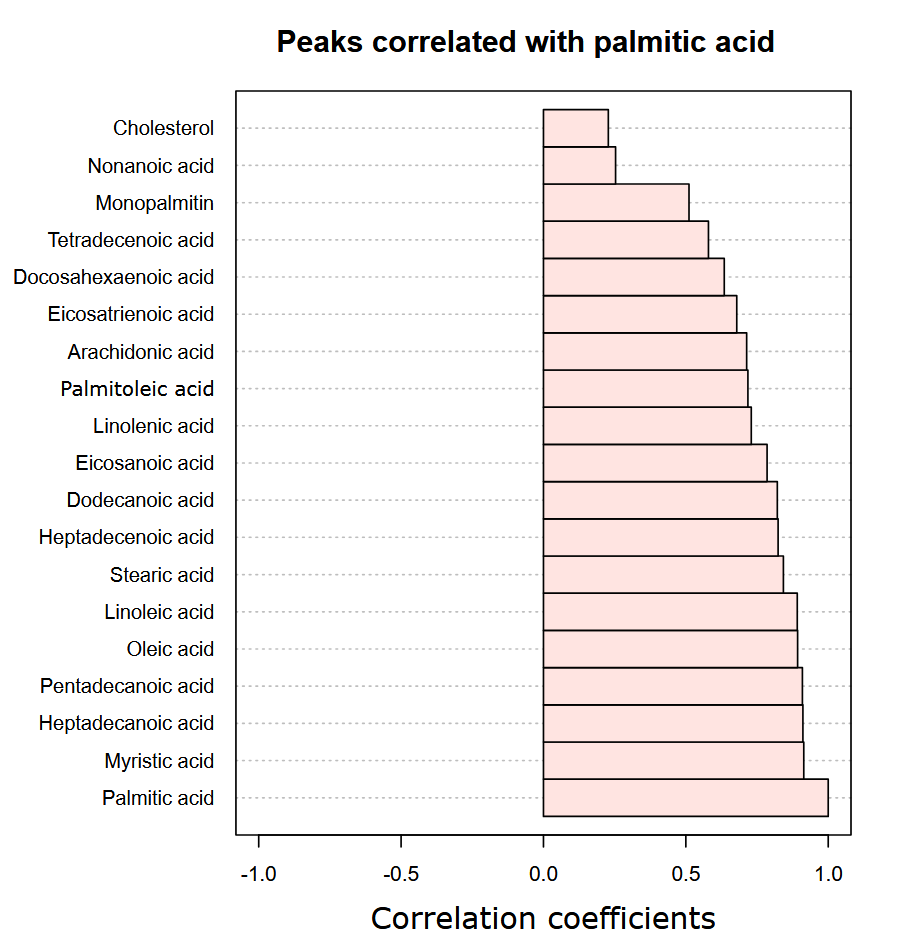


**Figure S5. Correlation Analysis of Quantified Fatty Acids.** This figure illustrates the co-enrichment of long-chain saturated fatty acids (e.g., stearic acid, monopalmitin, and heptadecanoic acid) with palmitic acid using Pearson correlation coefficients.


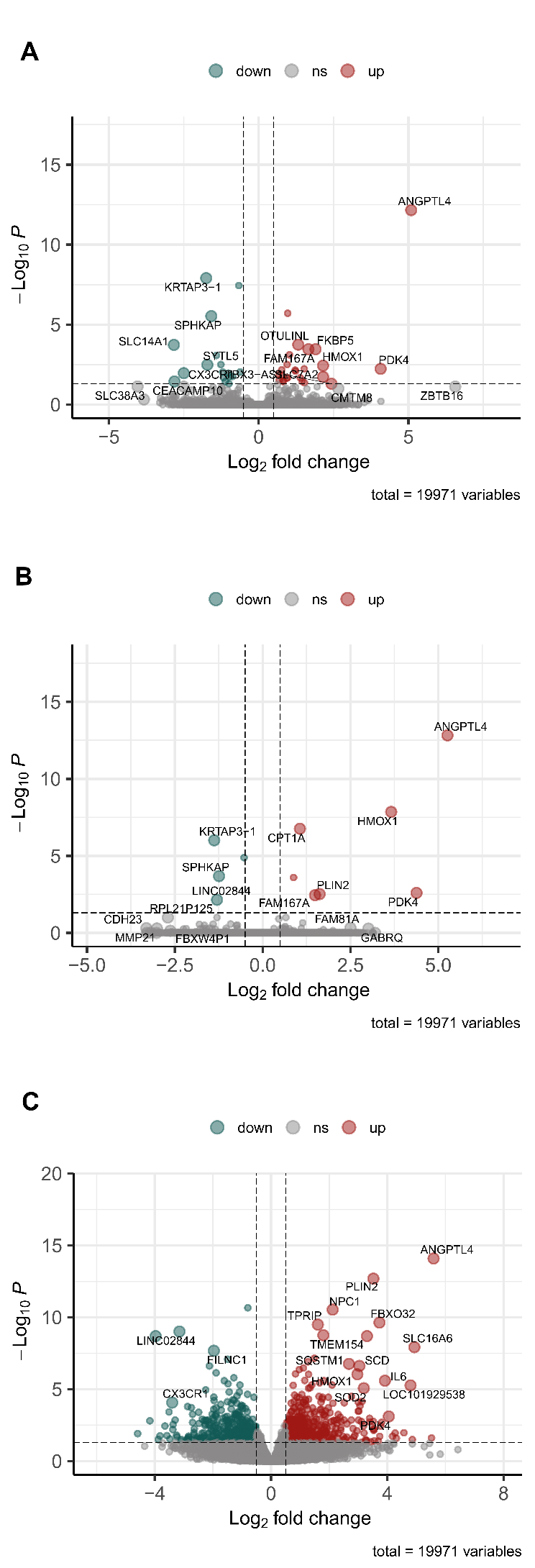


**Figure S6.** **CR-POPF effluents and monopalmitin engage a shared lipid-responsive core gene program in fibroblasts. A.** Cumulative volcano plot of differential gene expression in human foreskin fibroblasts (HFF) following exposure to AES1448 compared with control conditions. Data represent integrated results across independent fibroblast preparations. Red: significantly upregulated genes; green: significantly downregulated genes; grey: not significant (FDR < 0.05). **B.** Cumulative volcano plot for GR1479 compared with control. The gene-level distribution largely overlaps with AES1448, indicating engagement of a similar transcriptional core with limited overall amplitude. **C.** Cumulative volcano plot for monopalmitin (0.2 mM) compared with control. Monopalmitin induces a broader and higher-amplitude distribution of regulated genes compared with effluents, consistent with quantitative amplification of the shared lipid-responsive program. Overall transcriptional dispersion remains more constrained than observed in mesothelial cells.


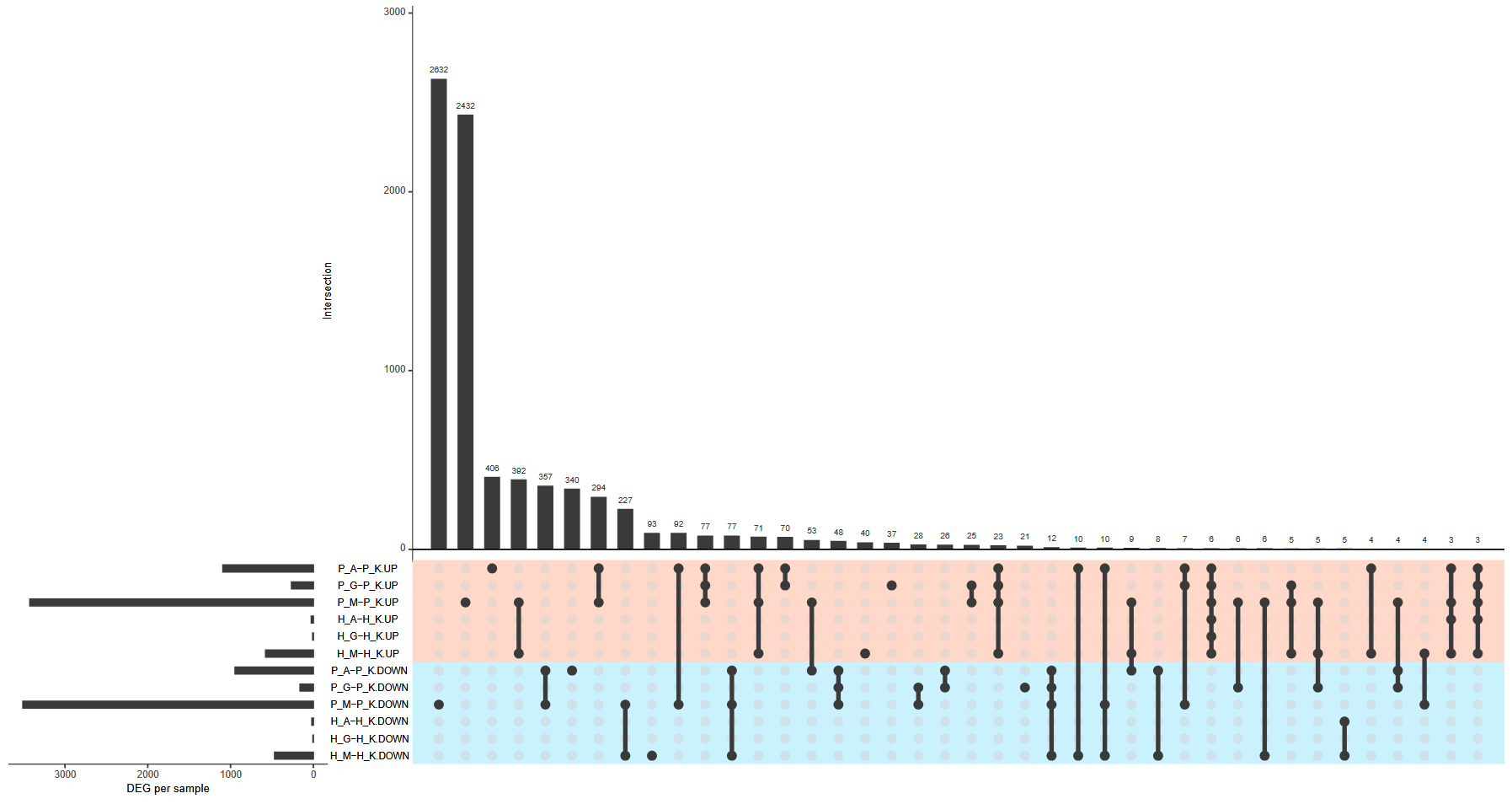


**Figure S7. UpSet plot of differentially expressed genes across stimuli and cell types**. Differentially expressed genes were defined as FDR < 0.05 and |log₂ fold change| > 1. Intersections of significantly upregulated (upper panel) and downregulated (lower panel) genes across peritoneal mesothelial cells and fibroblasts under AES1448, GR1479, and monopalmitin exposure are shown. Bars indicate intersection sizes, and horizontal bars depict total DEG counts per contrast. Most regulated genes were condition-specific, with limited universal overlap across stimuli and cell types.

**
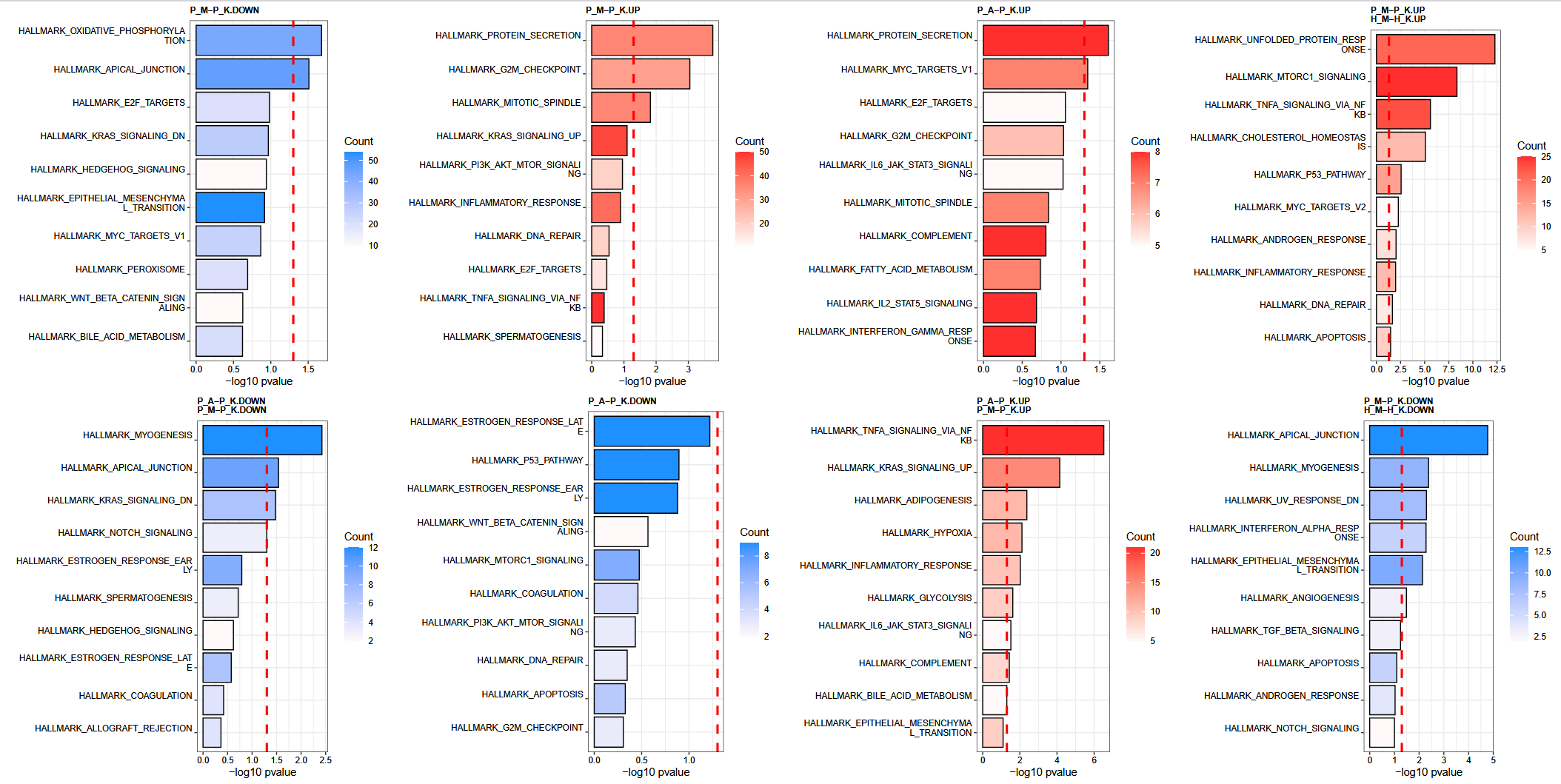
Figure S8A**

**Figure S8A-C. Overlapping hallmark gene sets across stimuli and cell types.** Comprehensive listing of hallmark gene sets contributing to shared and cell type–specific enrichment patterns as illustrated in Figure S7. Gene sets are grouped according to overlap between peritoneal mesothelial cells and fibroblasts under AES1448, GR1479, and monopalmitin exposure. Due to the large number of enriched gene sets, the complete listing is presented across multiple pages.

**Figure S8B**
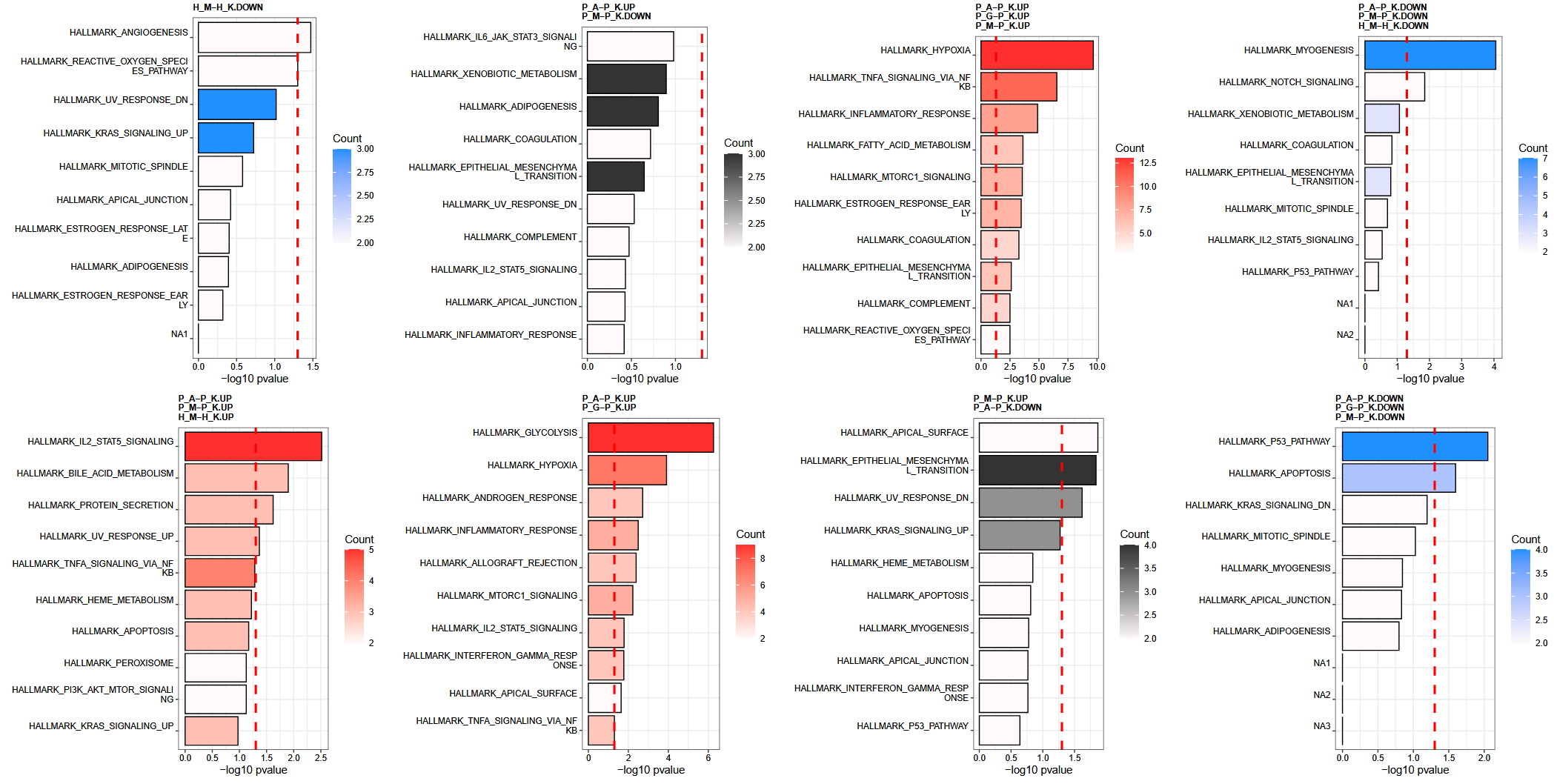


**Figure S8C**
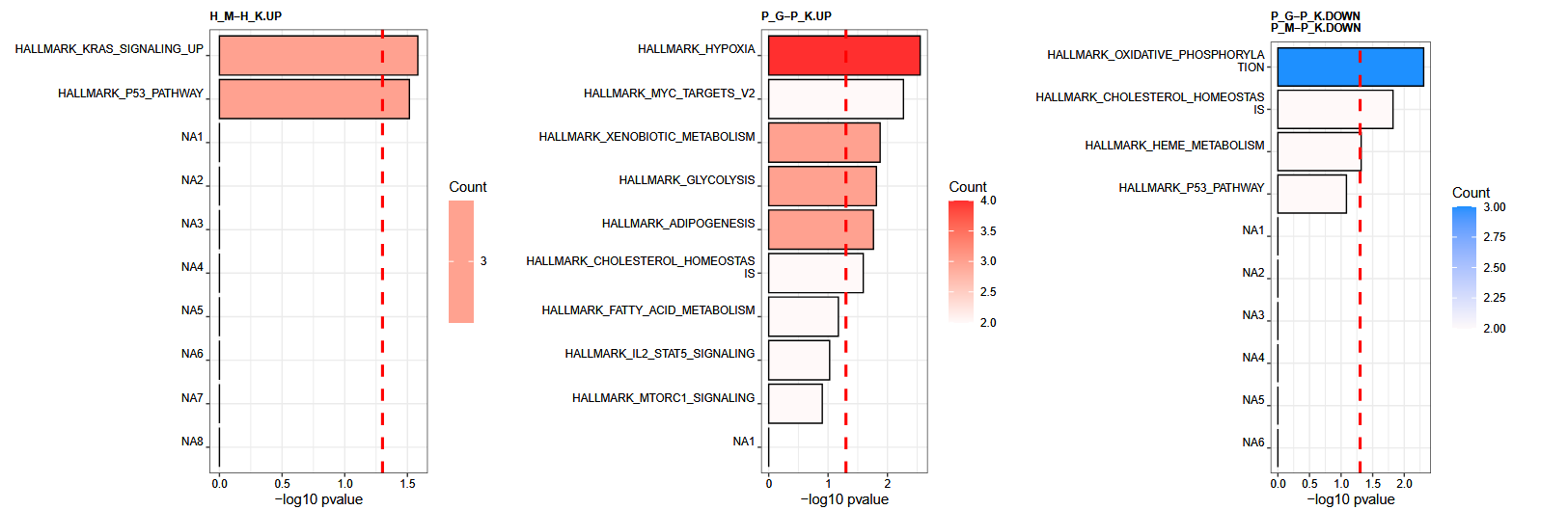


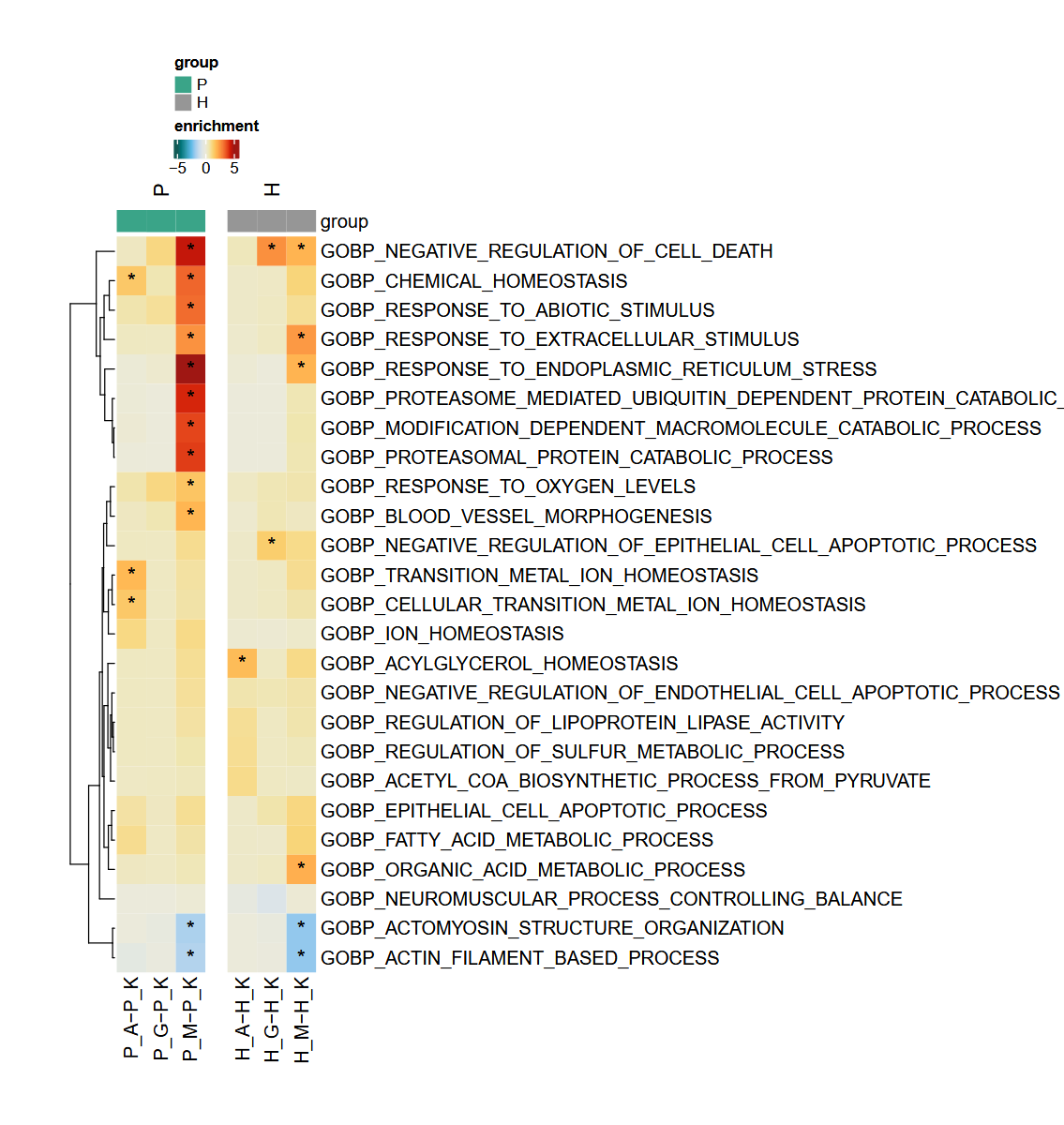


**Figure S9. GO Biological Process gene set enrichment across stimuli and cell types.**
Heatmap representation of significantly enriched GO Biological Process terms in peritoneal mesothelial cells (P) and fibroblasts (H) following stimulation with AES1448, GR1479, or monopalmitin compared with control. Color scale indicates normalized enrichment scores. All stimuli induced coordinated enrichment of stress-adaptive and metabolic processes, including endoplasmic reticulum stress, proteasome-mediated protein catabolism, and lipid metabolic pathways. Monopalmitin showed the highest enrichment magnitude across processes. Gene sets related to actin cytoskeleton organization demonstrated negative enrichment under monopalmitin exposure.


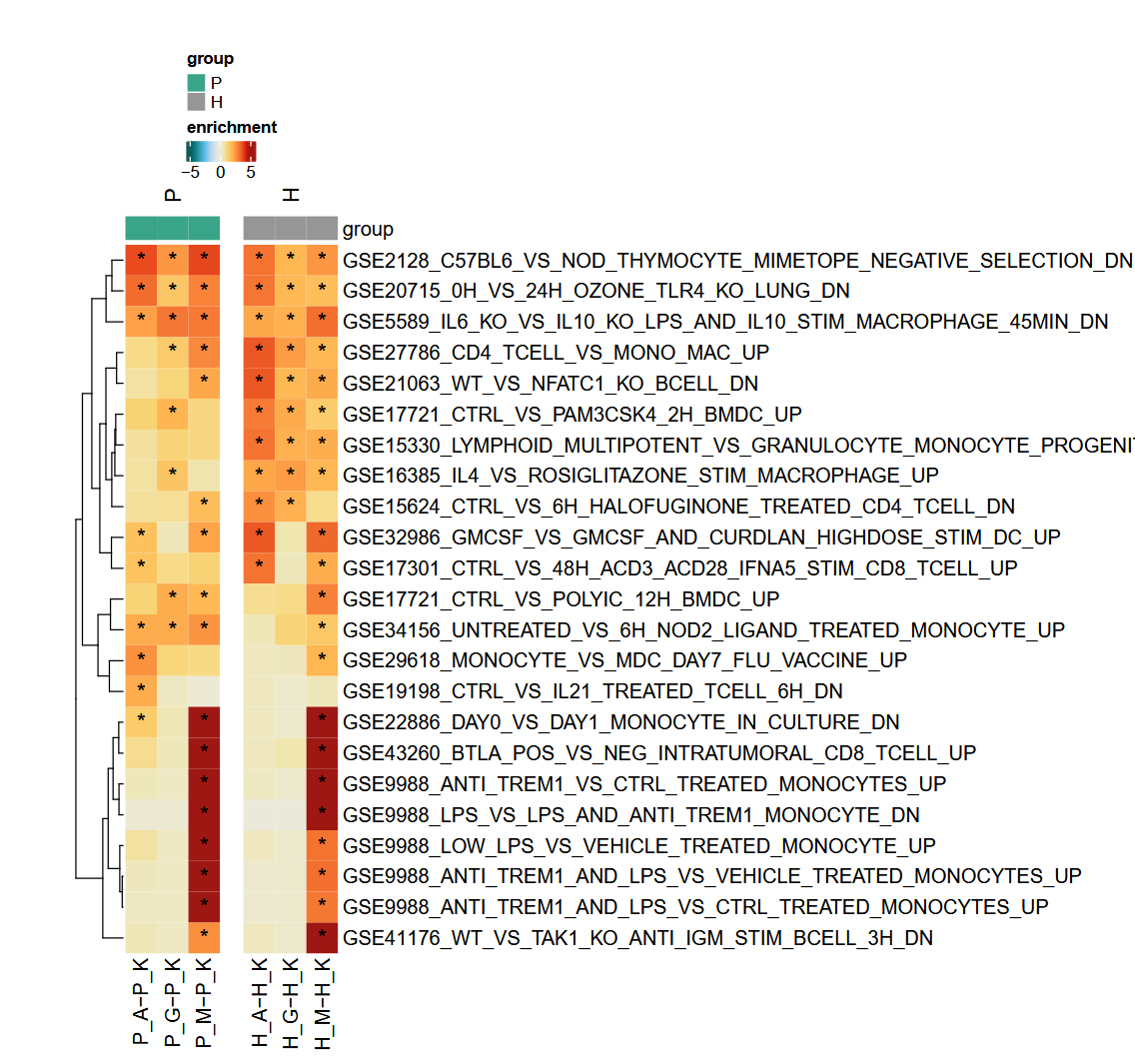


**Figure S10. C7 ImmunesigDB enrichment analysis across stimuli and cell types.** Heatmap showing normalized enrichment scores for selected ImmunesigDB gene signatures in peritoneal mesothelial cells (P) and fibroblasts (H) following stimulation with AES1448, GR1479, or monopalmitin compared with control. Enriched signatures include monocyte- and macrophage-associated activation datasets (e.g., PAM3CSK4-, NOD2 ligand-, and LPS-related stimulation profiles). Enrichment magnitude was highest following monopalmitin exposure and was more pronounced in peritoneal mesothelial cells compared with fibroblasts.


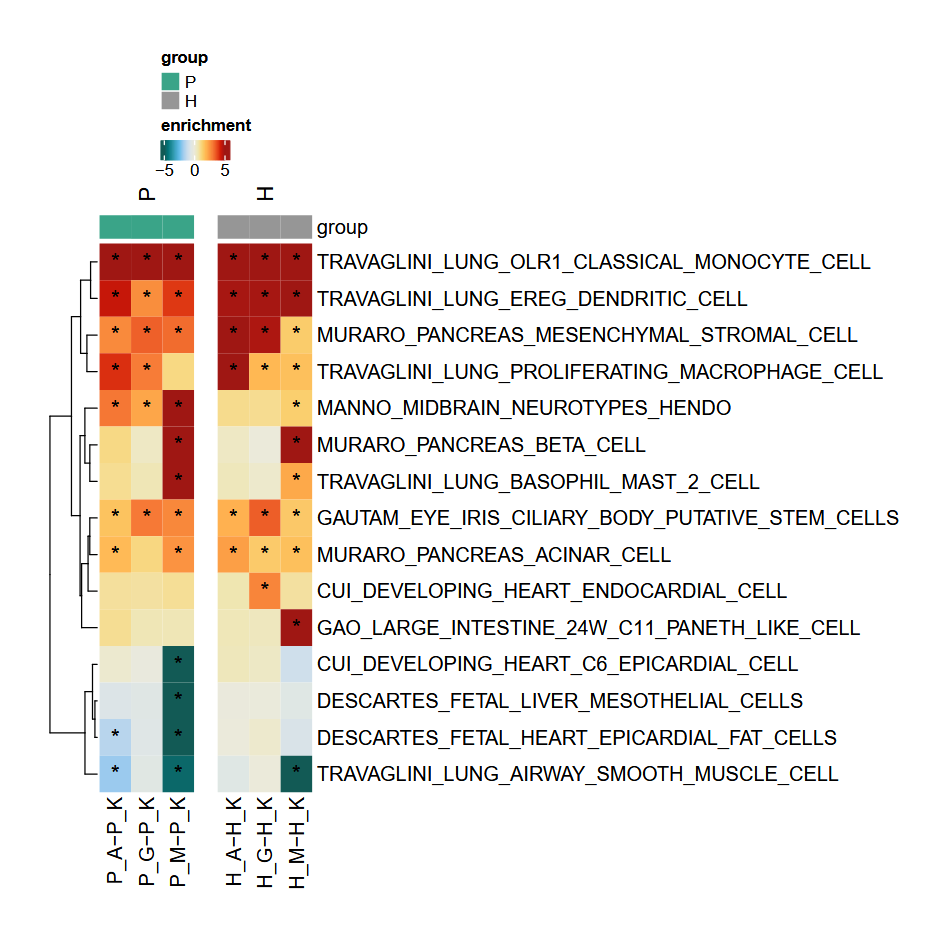
**Figure S11. C8 cell type signature enrichment analysis.** Heatmap displaying normalized enrichment scores for selected C8 cell type–associated gene signatures in peritoneal mesothelial cells (P) and fibroblasts (H) following stimulation with AES1448, GR1479, or monopalmitin compared with control. Enrichment of immune-associated cell signatures (e.g., monocyte, dendritic cell, macrophage datasets) was observed across stimuli, with higher magnitude in peritoneal mesothelial cells. Fibroblasts demonstrated comparatively greater representation of stromal-associated signatures. Enrichment intensity increased in a graded manner from GR1479 to AES1448 to monopalmitin.


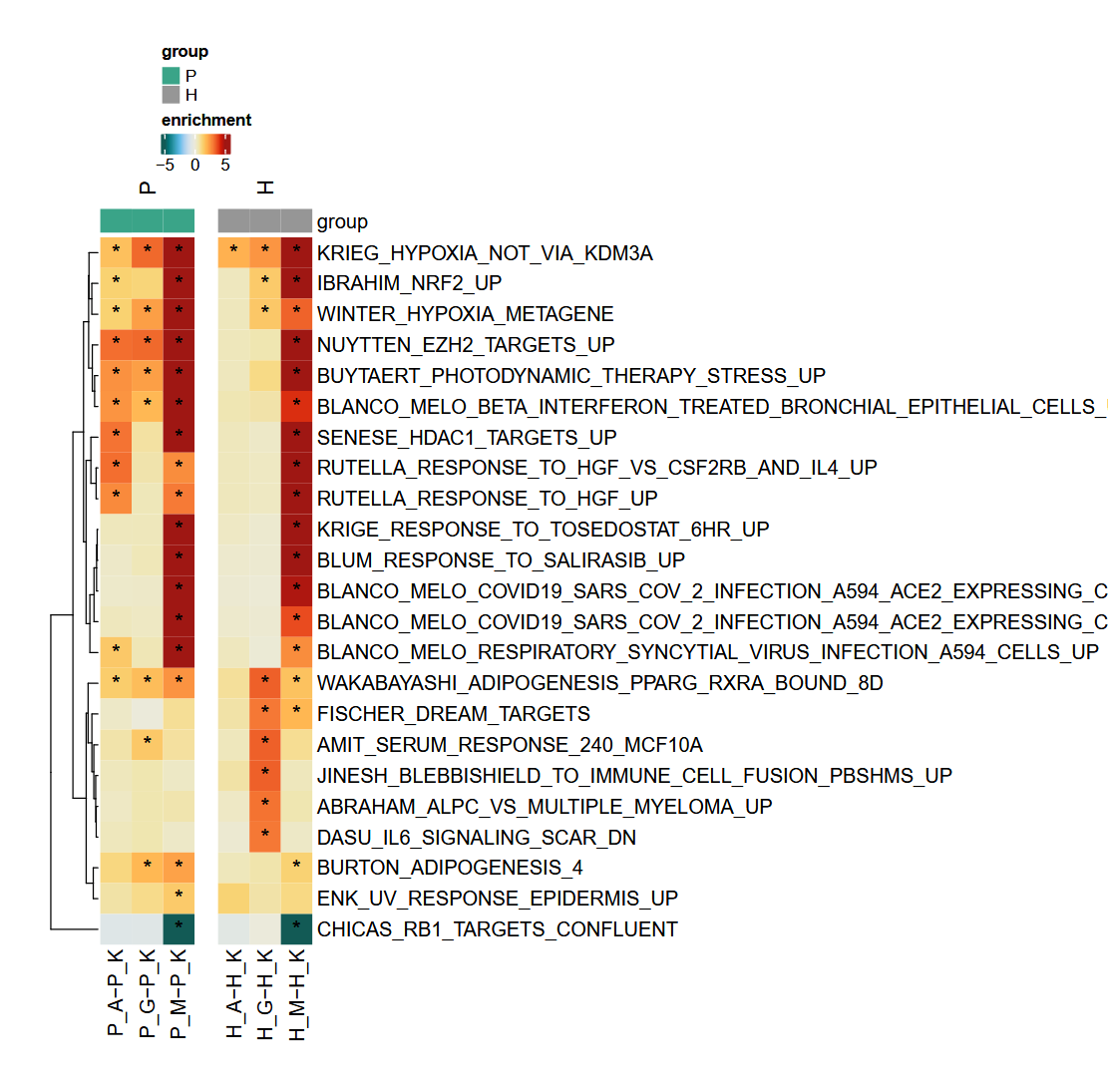


**Figure S12. C2–CGP (chemical and genetic perturbation) gene set enrichment analysis.**
Heatmap showing normalized enrichment scores for selected C2–CGP gene signatures in peritoneal mesothelial cells (P) and fibroblasts (H) following stimulation with AES1448, GR1479, or monopalmitin compared with control. Only gene sets with FDR-adjusted q < 0.05 are displayed. Enrichment of hypoxia- and oxidative stress–associated signatures (e.g., KRIEG_HYPOXIA_NOT_VIA_KDM3A, IBRAHIM_NRF2_UP, WINTER_HYPOXIA_METAGENE) was observed across stimuli, with the strongest magnitude following monopalmitin exposure. Peritoneal mesothelial cells demonstrated comparatively stronger stress-associated enrichment, whereas fibroblasts showed relatively greater representation of adipogenesis- and serum response–related signatures. Asterisks indicate FDR-adjusted significance
